# Transcriptomic functional characterization of recombinant adeno‐associated virus producing cell line adapted to suspension‐growth

**DOI:** 10.1002/btpr.70042

**Published:** 2025-05-21

**Authors:** Han‐Jung Kuo, Prahalad Srinivasan, Yu‐Chieh Lin, Min Lu, Carissa Rungkittikhun, Qi Zhang, Wei‐Shou Hu

**Affiliations:** ^1^ Department of Chemical Engineering and Materials Science University of Minnesota Minneapolis Minnesota USA

**Keywords:** gene therapy, recombinant adeno‐associated virus, synthetic biology, viral vector manufacturing technology

## Abstract

Recombinant adeno‐associated virus (rAAV) is a widely used delivery vehicle in gene therapy. A scalable production technology is essential for its wide clinical applications. We have taken a synthetic biology approach to generate HEK293‐based cell lines which harbor integrated genetic elements encoding essential AAV and adenoviral helper components and can be induced to produce rAAV. Through cycles of cell line enhancement, a high rAAV productivity could be achieved. The cell lines, like their parental HEK293, grew adherently. For scalable production, cell cultivation in suspension is highly desirable. A producer cell line GX6B was adapted to suspension growth in serum‐free medium (named GX6Bs). However, it had substantially reduced virus titer. Returning GX6Bs cells to adherent culture conditions using adherent medium and cultured stationarily brought the productivity back to close to the level of adherent GX6B. A survey of the transcriptome revealed that induction and rAAV production elicited a wide range of cellular changes in various functional classes, including host immune defense response and nucleosome organization. The response was more subdued in suspension‐growing GX6Bs. Upon reverting to adherent growth, the cellular transcriptome change regained its vigor to be more similar to that seen in GX6B. The GX6Bs maintained in suspension serum‐free conditions were then reverted to the adherent culture medium but under an agitated culture environment to keep suspension growth for rAAV production. The productivity returned to within 25%–50% of GX6B. This work demonstrated the feasibility of the suspension culture of synthetic cell lines for the expansion and production of rAAV.

## 1 INTRODUCTION

Adeno‐associated virus (AAV) is a small capsid virus with a 4.7 kb‐long single‐stranded DNA genome. The two genes in the genome, *rep* and *cap*, sandwiched in between two inverted terminal repeats (ITRs), generate six transcripts and nine proteins^1^ (Figure S1A). Its infection of a cell results in latent infection, and virus replication and release of infectious virus occur only when co‐infected by a helper virus, such as Adenovirus. For gene therapy applications, the viral genes in between two ITRs are replaced with a therapeutic gene to give rise to a recombinant AAV (rAAV) vector, which can be transduced to the target cell to enable them to produce the therapeutic gene product. The rAAV vector is safe since it cannot replicate by itself unless coinfection by a helper virus or helper genes is also provided. AAV has several serotypes with different tissue tropisms, is episomally stable for a long period after transduction into non‐dividing host cells, and is suitable for in vivo applications. It has become one of the most widely used in vivo gene delivery vehicles.^2^

rAAV is typically applied in very large doses in clinical applications; for systemic administration through intravenous injection, 10^13^–10^14^ physical particles per kg of patient's weight is not uncommon.^3^, ^4^ Such a large dose poses a major challenge to its manufacturing. Currently, rAAV is primarily produced by transient transfection of HEK293 cells with three plasmids containing the rAAV genome, the AAV *rep* and *cap* genes, and the adenoviral helper genes *E2A*, *E4*, and *VA RNA*. The genome of HEK293 encodes two helper genes, *E1A* and *E1B*, from Adenovirus. This method is simple but presents hurdles in large‐scale manufacturing, as it requires large quantities of multiple plasmids and large‐scale plasmid transfection.^2^ Others have produced rAAV using viral vectors based on the adenovirus, herpes simplex virus, or the baculovirus to provide the rAAV genome and AAV *rep* and *cap* genes.^3^, ^4^, ^5^, ^6^, ^7^ Upon co‐infection of these viral vectors, adenovirus rAAV is produced. However, this process requires additional downstream steps to ensure the removal of helper viruses and their nucleic acids and proteins.^8^

We developed a systems synthetic biology strategy and created stable cell lines with essential genetic elements integrated into their cell genomes whose induction set off rAAV production, thus eliminating the need for transfection with multiple plasmids or viruses.^9^, ^10^ Three genetic modules were integrated into the nuclear genome of HEK293 cells (Figure S1B): (1) a Genome Module (GM) encoding the cargo gene (in this case, a green fluorescent protein (GFP) reporter gene) flanked by AAV2 ITRs, (2) a Replication Module (RM) containing an inducible TetON promoter‐driven Rep68 coding sequence (CDS) and adenoviral helper E4orf6 and DBP CDS, and (3) a Packaging Module (PM) consisting of an inducible CumateSwitch promoter‐driven AAV2 intron‐less *cap* gene (VP123) and the Rep52 CDS.^9^, ^11^ The *rep* gene encodes four non‐structural Rep proteins: the large Rep proteins Rep78/68 are indispensable for AAV genome replication,^12^ while the small Rep proteins Rep52/40 are essential for AAV genome packaging.^13^ VP123 has been reported to give a better VP1:VP2:VP3 ratio that is closer to the 1:1:10 seen in capsids. Assembly‐activating protein (AAP), which facilitates capsid assembly,^14^ and membrane‐associated accessory protein (MAAP), which promotes AAV cellular egress but limits viral DNA replication,^15^, ^16^, ^17^ are also encoded by the VP123 gene. By replacing the native promoter of Rep68 and *cap* genes with inducible promoters, the design allowed the kinetics of expression of key components in the system to be modulated by the inducer concentration profile to optimize the productivity and quality of the rAAV produced.

The design was implemented through several cycles of design‐build‐characterization aided by transcriptomic analysis and targeted proteomic‐based viral protein absolute quantification. In the course of advancement, an inducible Lac promoter was adopted for the transcription of the cargo GFP gene to reduce its expression during rAAV2 production,^11^ and more copies of PM were integrated into the cellular genome to increase the production of VP proteins and capsids.^11^, ^18^

The stable cell lines generated by this strategy produced rAAV at titers on par with the conventional transient transfection‐based method.^18^ However, these producing cell lines, like their parental line HEK293, grew adherently. For industrial manufacturing on a large scale, suspension culture is preferred to adherent growth. As early as seven decades ago, BHK cells were adapted to suspension growth so large‐stirred tanks could be used to produce foot‐and‐mouth disease vaccine.^19^ To this date, cell lines like MDCK and Vero, commonly used in viral vaccine production, were adapted to grow in suspension culture for vaccine manufacturing.^20^, ^21^, ^22^ While adherent cells could be grown on microcarriers using stirred tanks, suspension cultures are far simpler for large‐scale operations. The CHO cells used in recombinant protein biologics production were originally adherent; they were adapted to suspension growth for manufacturing processes.^23^ Today, virtually all CHO cells used in recombinant protein production are all suspension‐growth adapted.

In this study, we adapted our GX6B cell line to grow in suspension under serum‐free conditions and saw a major reduction in its rAAV2 productivity. The adapted suspension cell line GX6Bs promptly reverted to adherent growth upon transferring to the original serum‐containing adherent medium and recovered its rAAV productivity to almost the original level. To better characterize this adherent‐suspension conversion, we employed RNA‐seq and targeted proteomics over the induction period. Herein we report the gene expression kinetics and insight gained from functional enrichment analysis.

## 2 MATERIALS AND METHODS

### 2.1 Cell and cell culture

The construction and characterization of the rAAV2 producer cell line GX6B were described previously^18^, ^24^ and are briefly described in Figure S1. The GX6B cell line was maintained in Dulbecco's Modified Eagle Medium (DMEM) with 4.5 g/L glucose (Gibco, ThermoFisher, Waltham, MA, USA) supplemented with 10% fetal bovine serum (FBS) (Gibco, ThermoFisher, Waltham, MA, USA) at 37°C in a humidified 5% CO_2_ air atmosphere. The medium for adapting GX6B to suspension growth and subsequent cell culture in suspension was a protein‐free Celer‐S001S medium (Bio‐engine, Shanghai, China) (kindly provided by Liang Zhao).

### 2.2 rAAV production

The rAAV producer GX6B was seeded at 4 × 10^5^ cells per well in 6‐well plates (7.8 × 10^4^ cells/cm^2^) using DMEM containing 10% FBS. After 16 h, the medium was replaced with fresh medium and induced with 10 μg/mL doxycycline and 90 μg/mL cumate (Sigma‐Aldrich, St. Louis, MO, USA). For rAAV production using GX6Bs, the cells were seeded at 4 × 10^5^ cells/mL in a shake flask with S001S medium and rotating at 100 rpm in an orbital shaker for three days before the cells were harvested and resuspended in S001S at 1 × 10^6^ cells/mL and induced with 10 μg/mL doxycycline and 90 μg/mL cumate. The cells were induced in 6‐well plates with shaking. Other induction conditions are described in the Supplementary Materials and Method section (Data S1).

For production using GX6Bs but under re‐adherent conditions (GX6BsR), the suspension cells were harvested on day 3 and seeded at 4 × 10^5^ cells/well in 6‐well plates using DMEM with 10% FBS for 16 h to allow for firm adhesion to the surface. The medium was then replaced with an induction medium containing 10 μg/mL doxycycline and 90 μg/mL cumate (Sigma‐Aldrich, St. Louis, MO, USA).

### 2.3 rAAV and viral component characterization

Sample preparation, rAAV titer measurement, RNA‐seq, and targeted proteomics are described in the Supplementary Materials and Method section (Data S1).

## 3 RESULTS AND DISCUSSION

### 3.1 Reduced productivity in serum‐free suspension culture

rAAV‐producing GX6B cells, grown adherently in DMEM with 10% FBS, were transferred to a serum‐free suspension medium, S001S, in a shaker flask. As described in Supplemental information (Figure S2A), after over four weeks, they became fully adapted to suspension growth. The suspension‐adapted cells, named GX6Bs, have a doubling time of around 33 h, somewhat longer than the 23 h of adherent GX6B cells in DMEM 10% FBS (Figure S2B,C). GX6Bs grew as genuine suspension cells with contrasting morphology to the parental adherent GX6B cells (Figure S2D,E). There was little cell aggregation, only a small degree of clumping.

**FIGURE 1:**
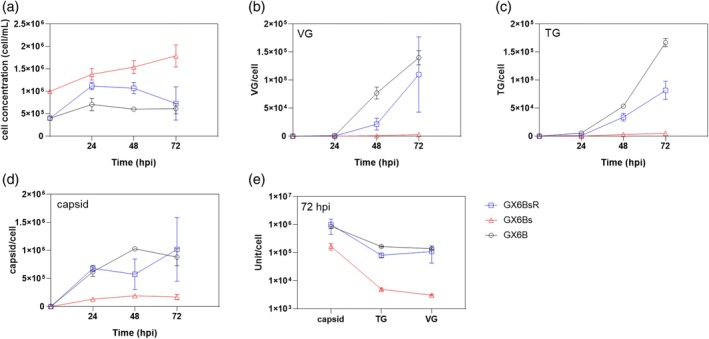
The kinetics of viral components production and cell growth in GX6B (black), GX6Bs (red), and GX6BsR (blue) after induction. (a) Cell concentration, (b) Encapsidated genome (VG) titer, (c) The total genome (TG) titer, (d) Capsid titer. All titers were normalized to per‐cell basis. The titer at 72 hpi was summarized (e). The error bar stands for SD, *n* = 3.

GX6B and GX6Bs cells at an early exponential growth phase were induced with 10D90C (10 μg/mL doxycycline and 90 μg/mL cumate) to allow for rAAV production. Typically, rAAV production by helper co‐infection or triple plasmid transfection results in extensive cell death after 48 h. In the case of rAAV production by induction of synthetic cell lines, the cell death is less prevalent at the time of virus harvest (48 or 72 h). There was even some increase in cell number (Figure 1a, for morphology Figures S2D,E and S3A,B).

At 72 h post induction (hpi) GX6B cells were harvested, and titers were quantified in terms of total rAAV genomes (TG), capsids, and encapsidated genomes (VG) and reported on titer per cell based on the cell concentration at the time of harvesting. GX6B produced its typical titer of TG and VG of somewhat higher than 10^5^ particles/cell, while GX6Bs cells produced only 3 × 10^3^ VG/cell (Figure 1b–d) as well as substantially lower levels of TG and capsids. The time profile showed that after induction, the assembled capsids in both GX6B and GX6Bs reached near peak value in 24 h (Figure 1d), while the production of viral genome and packaging of genome into capsid (to form VG) lagged behind. The final VG titer and TG titer for GX6B were similar (about 1.5 × 10^5^/cell), indicating that the packaging of viral genome into capsids was efficient.

### 3.2 Reversal of GX6Bs to adherent growth recovered most of the lost productivity

We observed that GX6Bs cells, upon transferring to tissue culture plates with the adherent medium (DMEM supplemented with 10% FBS) used to culture GX6B, reverted promptly to the adherent morphology resembling GX6B (Figure S2F). The re‐adherent GX6Bs had a doubling time of 26 h, similar to GX6B (Figure S2G). Herein, the case of GX6Bs grown under adherent conditions is referred to as GX6BsR. The faster growth rate was likely the result of the combined effect of adherent growth and the presence of serum in the medium.

To test whether the reversal of GX6Bs to adherent growth would revert the productivity to the level of its parental GX6B, GX6Bs cells grown in suspension were transferred to a stationary tissue culture plate surface with DMEM medium with 10% FBS. Sixteen hours after switching to adherent growth conditions, they were induced for rAAV production. The morphology at 48 hpi did not reveal extensive cell death as in infection or transfection‐based rAAV production (Figure S3C). The VG, TG, and capsid titers at 72 hpi restored to similar or somewhat lower levels than the original GX6B (Figure 1b–e), and so did the post‐induction cell growth (Figure 1a).

### 3.3 Viral component expression

**FIGURE 2:**
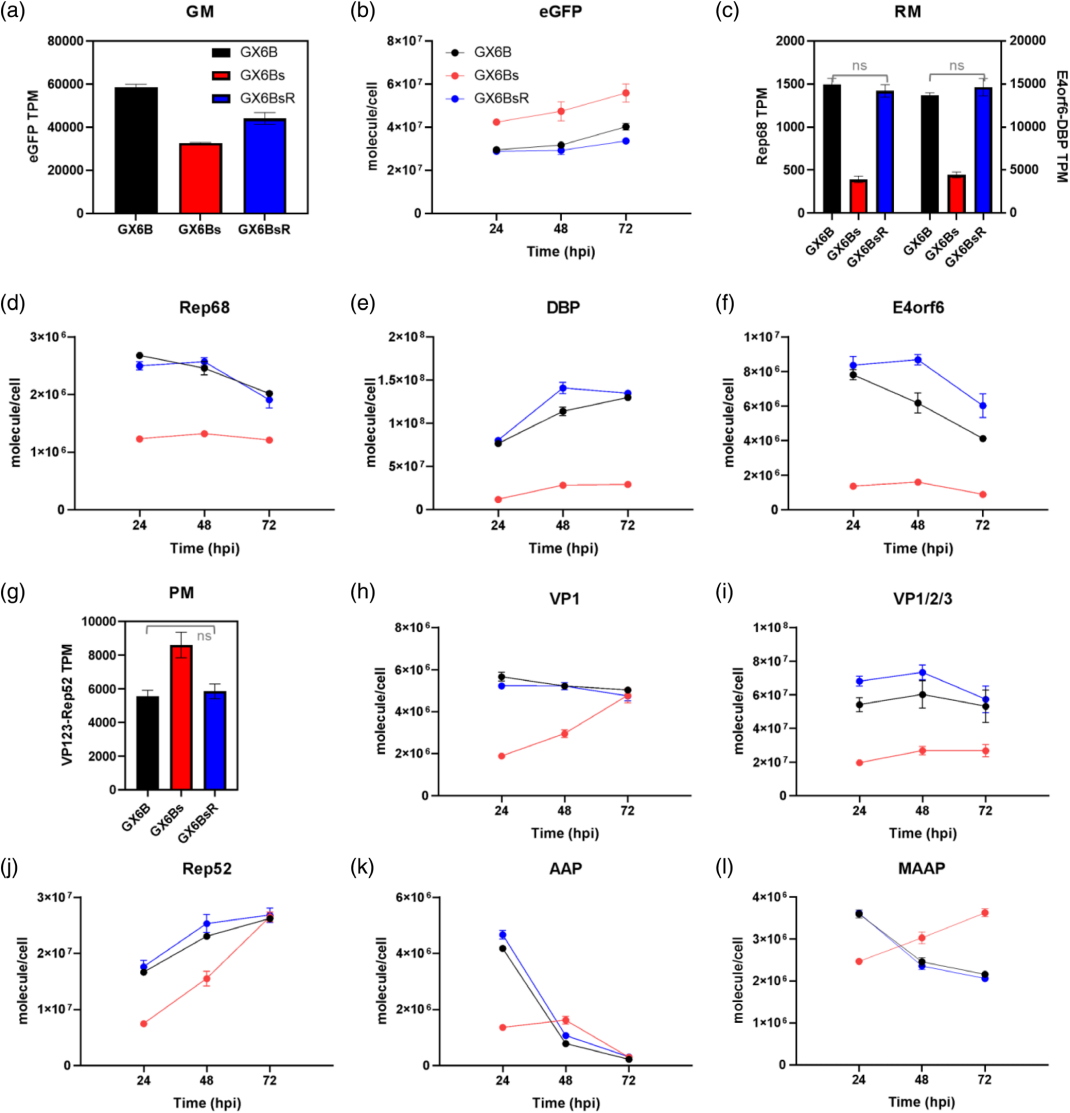
The viral transcripts and the viral proteins in GX6B (black), GX6Bs (red), and GX6BsR (blue) after induction. The viral genes encoded by GM (a, b), RM (d–f), and PM (g–l) were shown separately. (a), (c), and (g) were TPM (at 48 hpi) of transcripts encoded in each module, eGFP represents transcripts of GM (a), RM generates two transcripts DD‐mCherry‐Rep68 transcript and E4orf6‐(P2A)‐DBP transcript, each represented by Rep68 (c) and E4orf6‐DBP (g) respectively. VP123‐Rep52 represents VP123‐IRES‐Rep52‐(P2A)‐RFP transcript of PM. (d–f) and (h–l) were the time‐dynamic protein levels measured by AQUA. The error bar stands for SD, *n* = 3.

We investigated the viral gene expression at transcript and protein levels after induction in GX6B, GX6Bs, and re‐adherent GX6Bs (denoted as GX6BsR). The transcriptome, obtained by RNA‐seq and expressed as TPM (transcript per million), was obtained at 0 and 48 hpi, while viral proteins, quantified by targeted proteomic absolute quantification (AQUA) assay, were performed on samples of 24, 48, and 72 hpi and presented as the number of molecules per cell. Our earlier studies have shown viral transcripts largely stayed at similar levels after 48 hpi, and viral protein levels could have more dynamic behavior.^25^, ^26^ The results, shown in Figure 2, are grouped by the genetic module in which the coding sequence (CDS) is encoded.

The eGFP transcript encoded in the genome module was very high, about 60,000 TPM in GX6B (Figure 2a). It reduced to about half in GX6Bs but reverted to a higher level under re‐adherent conditions, the GX6BsR. The very high transcript level reflected the very high genome copy number after induction, especially in GX6B and GX6BsR (Figure 1c). Surprisingly, at the protein level, eGFP was higher in GX6Bs (Figure 2b) than in the other two cases. Importantly, GX6B and GX6BsR behave more similarly to each other than to GX6Bs.

The Replication module encodes two transcripts: one consists of E4orf6‐(P2A)‐DBP and the other consists of mCherry‐Rep68 fusion protein. Even though the two transcripts were both under TetON promoter control, the E4orf6‐(P2A)‐DBP transcript was expressed nearly 10‐fold higher than the Rep68 transcript (Figure 2c). TPM of both Rep and E4orf6‐DBP transcripts was high in GX6B, reduced substantially in GX6Bs, and mostly restored to a high level in GX6BsR (Figure 2c). The transcript profile of Rep68 and DBP of the three conditions was confirmed by qRT‐PCR (Figure S4A,B).

The two transcripts encoded in RM are translated into three proteins: Rep68, DBP, and E4orf6. The expression of these three proteins followed their transcripts, very high in GX6B, diminished in GX6Bs, and returned to a high level in GX6BsR (Figure 2d–f). Interestingly, even though DBP and E4orf6 were on one single transcript, DBP proteins were at levels of 10^8^ molecules/cell as compared to 10^7^ molecules/cell for E4orf6 (Figure 2e,f) suggesting translational regulation plays a role in their expression.

The three proteins Rep68, DBP, and E4orf6 play key roles in genome replication.^27^ All three proteins were expressed nearly an order of magnitude lower in the suspension GX6Bs cells (Figure 2d–f). Their lower expression in GX6Bs coincides with the lower TG productivity of GX6Bs. Whether the reduced expression of those proteins contributed to TG and VG productivity awaits further elucidation.

The intron‐less VP123 used in PM has been engineered to tune the ratio of VP proteins.^11^ The viral genes VP123 and Rep52 are encoded as a single transcript in PM and linked together by an IRES sequence. The transcript level, as represented by the average of TPMs of the whole transcript, from VP123 to RFP (Figure S1B), was higher in GX6Bs than in GX6B and GX6BsR as measured by both RNA‐seq and qPCR (Figures 2g, S4C). On the expression of genes encoded in PM, we again saw the same trend of GX6B and GX6BsR behaving similarly at both transcript and protein levels. However, in this case, GX6Bs was higher than GX6B and GX6BsR at the transcript level but lower at the protein level (Figure 2h–j). The divergence of transcript and protein levels suggested that yet to be elucidated viral gene expression control at transcriptional and translational, maybe even posttranslational levels. Since in our modular design the native viral regulatory promoters were replaced by inducible promoters, the data also suggested that there might be additional unidentified regulatory sequences in the CDSs in PM module. For AQUA analysis of VP1, a unique peptide was available. However, no unique peptide was available to differentiate the overlapping region of VP1, VP2, and VP3. Hence the measured value quantifies the total of all three and was designated as VP1/2/3. The ratio of VP1: VP2: VP3 proteins in AAV capsid is 1:1:10.^28^ In all three cases, GX6B, GX6Bs, and GX6BsR, the VP1 was nearly ten times lower than VP1/2/3 at 72 hpi as we had intended when we chose to use VP123 instead of cap CDS.^29^ The *cap* gene (VP123) in PM encodes two other proteins, AAP and MAAP. The protein profile of VP1/2/3, Rep52, AAP, and MAAP all showed again the similarity between GX6B and GX6BsR and the deviation of GX6Bs from them (Figure 2k,l).

### 3.4 Transcriptome dynamics of GX6B, GX6Bs and re‐adherent GX6BsR

#### 3.4.1 Quality of transcriptome data (PCA)

The transcriptome of GX6B, GX6Bs, and GX6BsR at 0 and 48 hpi was acquired by RNA‐seq. Principal component analysis (PCA) clustered each triplicate sample together in the PC1 versus PC2 plot as expected (Figure S5). Using the pre‐induction (0 hpi) samples as the reference (the denominator), differential expression analysis was performed on transcriptomes of 48 hpi (Figure S6). The criteria for differential expression call were |log_2_FC| > 1 (greater than two‐fold change in transcript abundance) and FDR <0.05. In both GX6B and GX6BsR, the induction caused many genes to differentially express; over 4000 genes were differentially expressed at 48 hpi, most of them upregulated. In contrast, the response of GX6Bs to induction and rAAV production was more subdued, with only around 1000 DEGs. Like in the other two cases, the vast majority of those DEGs were upregulated.

#### 3.4.2 Hierarchical clustering

**FIGURE 3:**
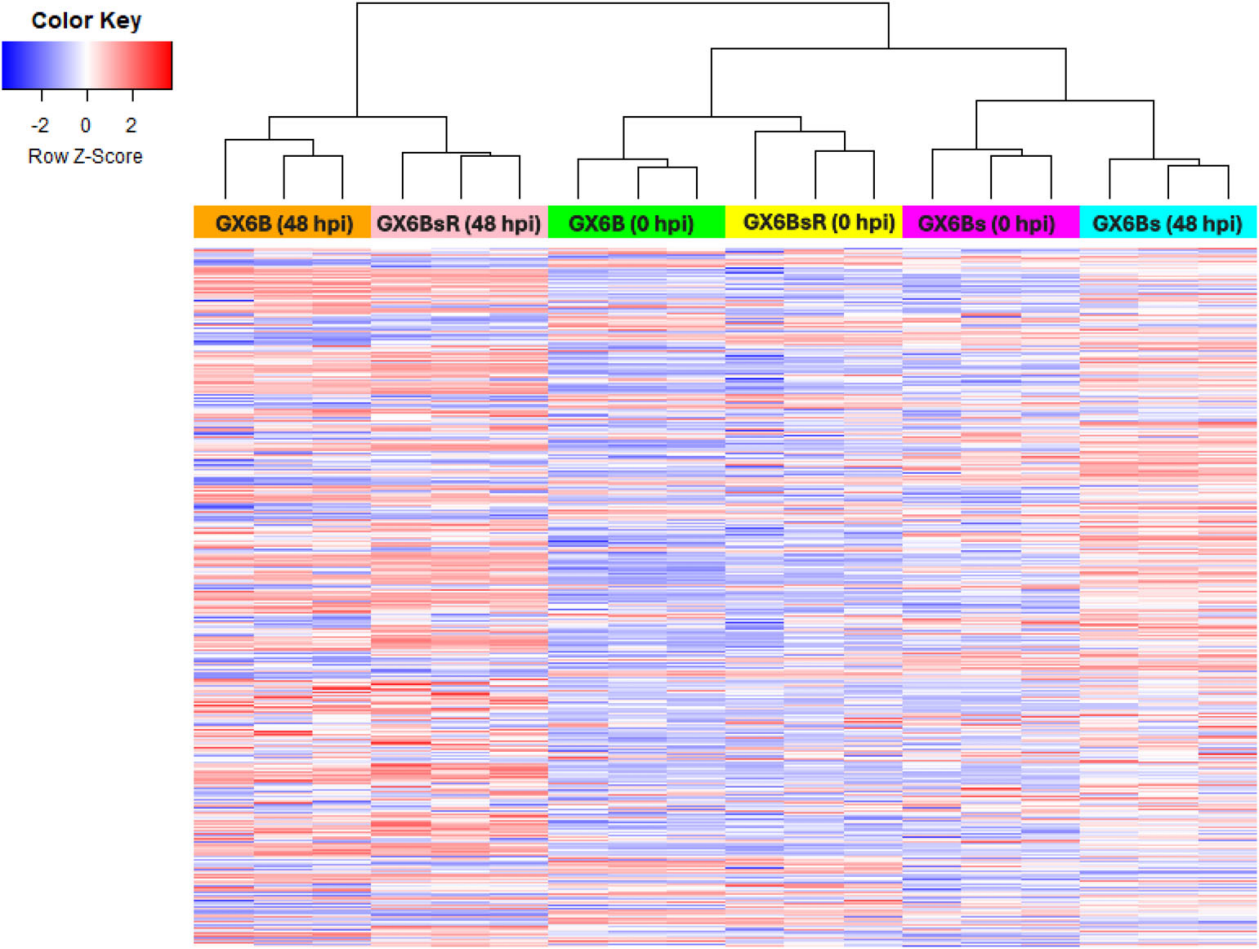
The hierarchical clustering heatmap across all samples and time points. Biological replicates for a sample are clustered together first. GX6B and GX6BsR are more similar to each other for a given time point, compared to GX6Bs. The GX6Bs 48 hpi sample being clustered with 0 hpi time points for all samples potentially indicates there is very little gene expression change at 48 hpi (compared to GX6B and GX6BsR).

The abundance levels (count per million, CPM) of all expressed genes on all samples were subjected to hierarchical clustering and shown as a heatmap (Figure 3). As expected, all biological replicates were grouped together. The GX6B (0 hpi) and GX6BsR (0 hpi) were clustered together first, and so were GX6B (48 hpi) and GX6BsR (48 hpi). Interestingly, GX6Bs (0 hpi) and GX6Bs (48 hpi) formed a cluster next. This cluster was then clustered together with the uninduced (0 hpi) sample of GX6B and GX6BsR, suggesting that the induction (48 hpi) in GX6Bs did not cause as much change in transcriptome as in GX6B and GX6BsR. The clustering results showed that GX6Bs, upon re‐adhering to surface, became more similar to GX6B than to itself in a suspension state. In short, both clustering and differential expression analysis of the transcriptome show that GX6Bs returned to a state similar to GX6B once resumed adherent growth.

### 3.5 Functional classes enriched in induction

**FIGURE 4:**
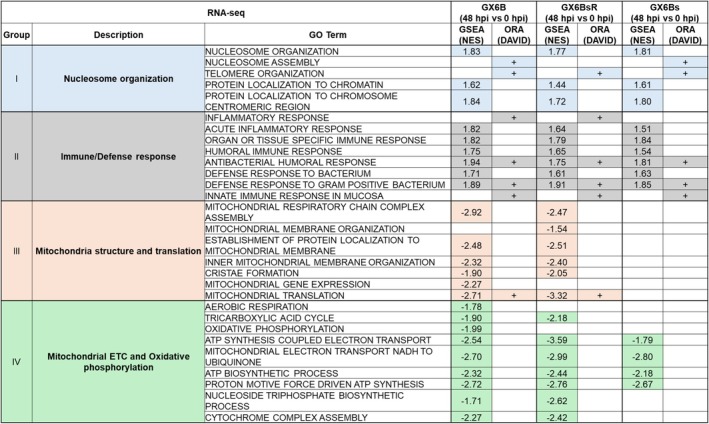
Functional groups of GO terms identified as enriched by ORA or GSEA after induction.

All DEGs (both up and down‐regulated) in each of the three pairs of 48 hpi/0 hpi comparison were subjected to overrepresentation analysis (ORA) using DAVID to identify the GO terms of Biological Process that were overrepresented. The enriched functional classes were then clustered using DAVID. Two clusters of GO terms related to nucleosome organization and immune/defense response were common to at least two of the three pairs of 48 hpi/0 hpi comparisons (Figure 4).

GSEA, which used the abundance level of all transcripts as inputs instead of only DEGs as in ORA, was also performed to identify enriched functional gene sets using the same GO terms as gene sets. GSEA used a different method to assess gene set enrichment and hence complements ORA using DAVID. Two GO terms (Antimicrobial humoral response and Defense response to Gram‐positive bacterium) identified as over‐represented by DAVID in Cluster II were also identified as enriched by GSEA. Overall, GSEA called more GO terms as enriched than ORA. Although the number of GO terms identified as enriched by both ORA and GSEA was small, many enriched GO terms identified by GSEA were functionally related to nucleosome organization or immune/defense response. Those GSEA‐enriched GO terms of relevant functions are also shown in Figure 4 in Group I or II. The fact that ORA and GSEA identified functionally overlapping GO terms supports the notion that these functions underwent changes upon induction. The three pairs of 48 hpi/0 hpi comparison for GX6B, GX6Bs, GX6BsR revealed a similar enrichment pattern.

In addition to GO terms in Group I and Group II, GSEA also identified several enriched and down‐regulated GO terms related to mitochondrial structure and translation, and mitochondrial electron transport chain (ETC) and oxidative phosphorylation in GX6B and GX6BsR. The Normalized Enrichment Score (NES) for those GO terms did not meet the cut‐off value for GX6Bs. The ORA for those mitochondria‐related GO terms did not call them as enriched. Our previous study using another synthetic cell line GX2 also showed similar down‐regulation of those mitochondria‐related terms 48 h after induction.^25^ The results again support the notion that the overall response in GX6B and GX6BsR was similar.

#### 3.5.1 DEGs in enriched function classes have high similarity in GX6B and GX6BsR

**FIGURE 5:**
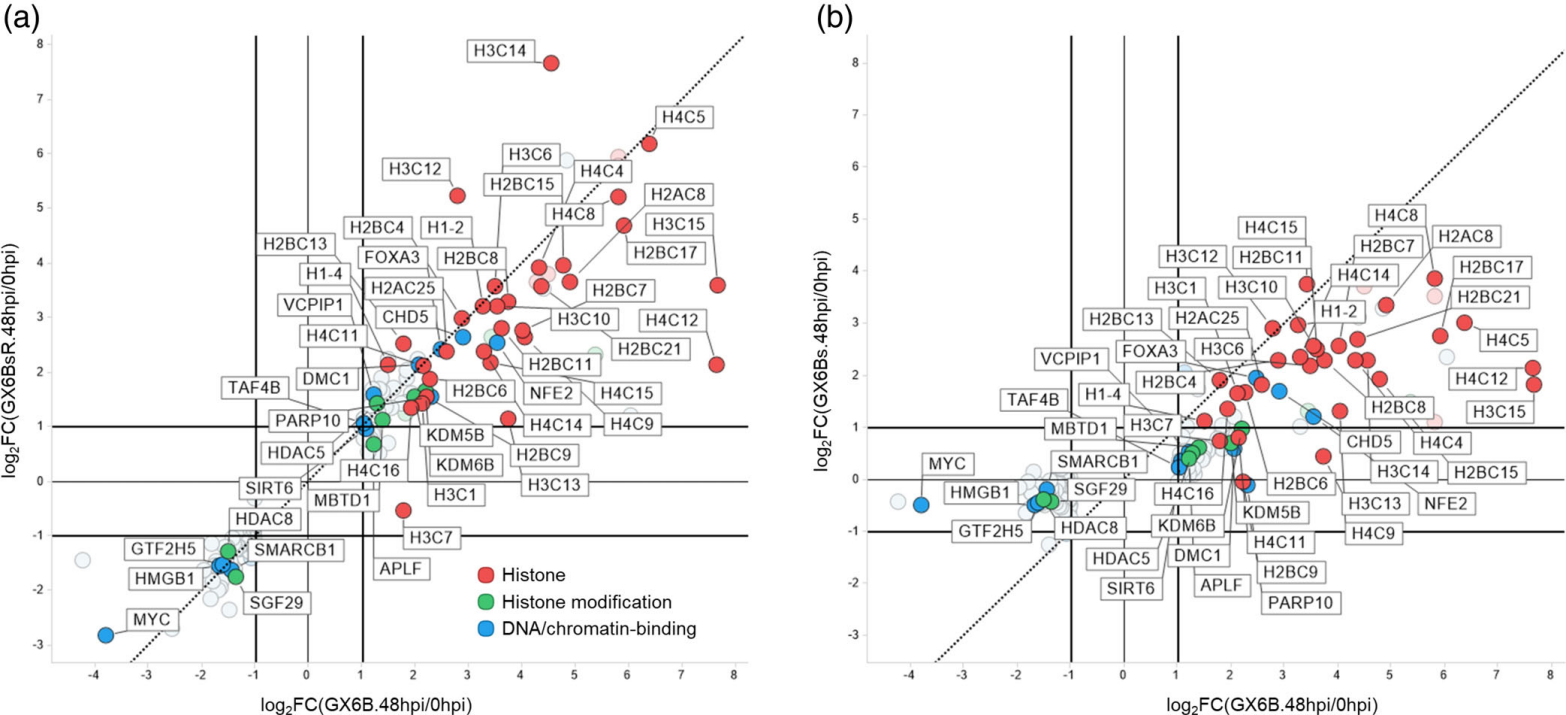
A double differential expression plot of genes in Group I nucleosome organization. Only DEGs at 48 hpi in GX6B are shown. The fold change of the genes after induction in GX6B (log2FC(GX6B.48hpi/0hpi)) was compared to GX6BsR (log2FC(GX6BsR.48hpi/0hpi)) (a) and GX6Bs (log2FC(GX6Bs.48hpi/0hpi)) (b). The dashed line represents the x = y line. The genes were listed in Table S1 and grouped into 3 groups based on their functions: Histone proteins (red), histone modification proteins (green), and DNA or chromatin‐related proteins (blue).

**FIGURE 6:**
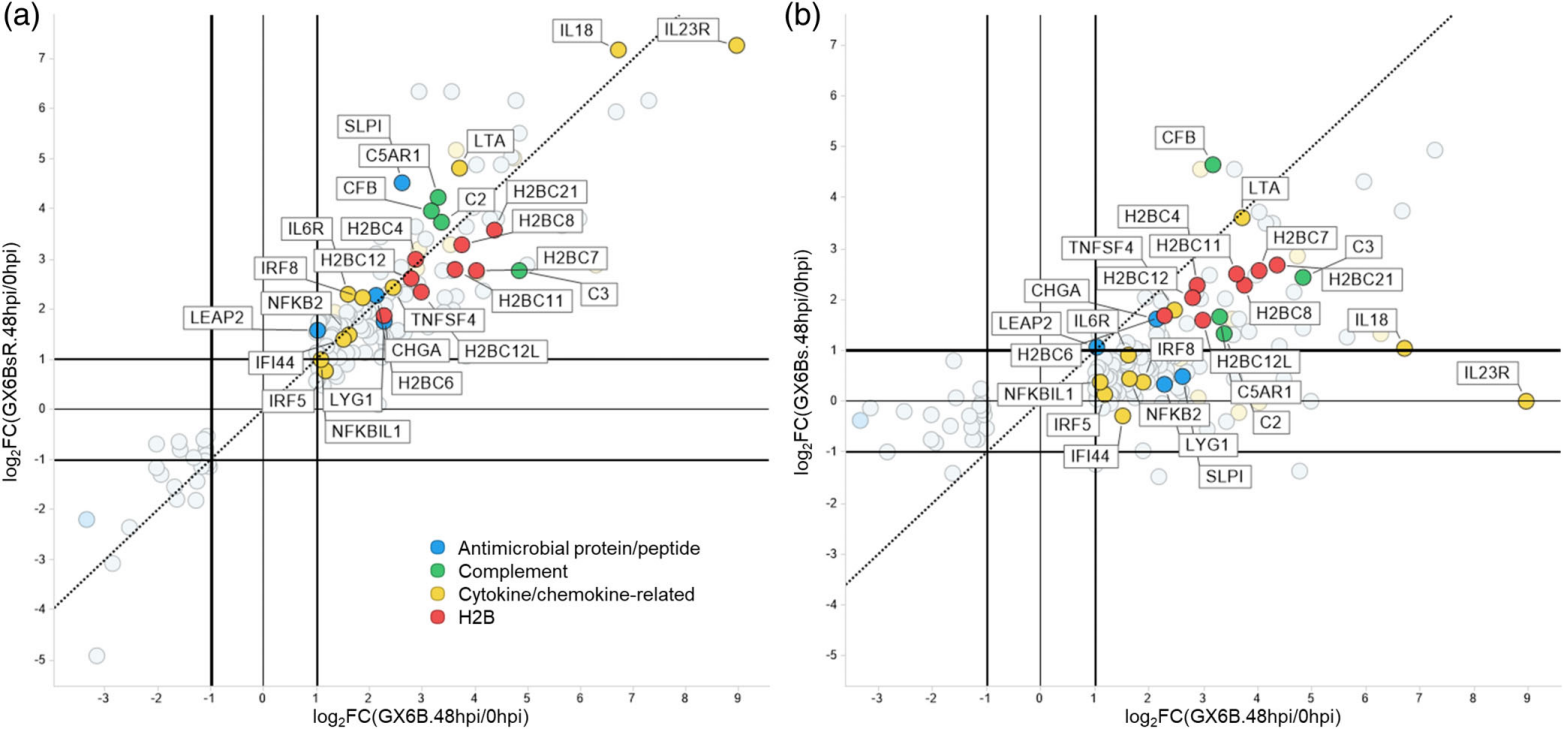
A double differential expression plot of genes in group II, Immune/Defense response, Only DEGs at 48 hpi in GX6B are shown. The fold change of the genes after induction in GX6B (log2FC(GX6B.48hpi/0hpi)) was compared to GX6BsR (log2FC(GX6BsR.48hpi/0hpi)) (a) and GX6Bs (log2FC(GX6Bs.48hpi/0hpi)) (b). The dashed line indicates the x = y line. The genes were listed in Table S2 and were grouped into 4 groups: Antimicrobial proteins or peptides (blue), complement (green), cytokine or chemokine‐related proteins (yellow), and H2B (red).

In GX6B(48/0 hpi) comparison, 154 and 151 DEGs belonged to GO terms in Groups I and II, respectively. The log_2_(fold change) of these DEGs was plotted in Figures 5 and 6 with GX6B as the common reference on the x‐axis and GX6BsR (Figures 5a, 6a) or GX6Bs (Figures 5b, 6b) on the y‐axis. The concordance of the transcript expression profile upon induction between GX6B and GX6BsR was evident (Figures 5a, 6a). These DEGs were differentially expressed in the same direction of up‐ or down‐regulation in GX6B and BX6BsR. The magnitudes of fold change (Figure 5a) of most genes were very similar between the two cases, as most of the DEGs lined up along the 45° (x = y) line in the plot. Few DEGs resided in Quadrant II and IV where genes were up‐ or down‐regulated in opposite directions.

Comparison of GX6Bs and GX6B (Figures 5b, 6b) revealed a similar trend of differential expression. The vast majority of DEGs changed in the same direction. However, for GX6Bs, the extent of expression level change was lower than that of GX6B, as seen in Figures 5b and 6b; many points in quadrant I shifted downward to the right of the x = y line. At the gene level, most DEGs in those functional groups in GX6Bs and GX6BsR responded in the same direction as GX6B. However, in terms of the magnitude of differential expression, GX6Bs had a more tempered response to induction than GX6B, while in re‐adherent GX6BsR, the degree of differential expression returned to the higher level similar to that of GX6B.

The gene symbols of some representative genes in Figures 5 and 6 are labeled and listed in Tables S1 and S2. In Group I of Nucleosome organization, many DEGs fall into histone proteins (such as H1, H2B, H3, and H4), histone modification proteins (such as KDM6B, KDM5B, and MBTD1) and other DNA‐binding or chromatin‐related proteins (such as transcription factor, helicase, and chromatin‐binding proteins). Some transcription regulators, such as MYC, NFE2, and FOXA3, were involved in many cell development processes.^30^, ^31^, ^32^ Histone and nucleosome reorganization contribute to the epigenetic regulation of transcription, chromosome condensation, and DNA repair. Upon virus infection or induction, the virus production process, as in our case, transcriptional activities increased drastically, which surely would involve epigenetic alteration of genome accessibility. Additionally, tremendous amplification of the rAAV genome occurs in all cells. The upregulation of histone proteins stabilizes the huge number of copies of the viral genome. The induction causes the regulation of nucleosome‐related proteins to be synchronized in all cells, making the upregulation even more prominent.

A group of upregulated histone genes related to H2B was also grouped in GO terms related to Response to bacterium and was grouped together in Group II. H2B is not only involved in chromatin structure but also presents extrachromosomally as cytosolic sensors to detect double‐stranded DNA from microbial infection or cell damage.^33^ H2B thus plays an important role in the innate immune response.^34^ Several genes in this functional group have antimicrobial or antiviral activities, including those encoding complement C2/C3, LEAP2, and LYG1, which have activities against Gram‐positive bacteria,^35^, ^36^ and CHGA encodes an antibacterial peptide.^37^, ^38^ Many genes in Group II are involved in cytokine or chemokine signaling, including several interleukins and their receptors, such as IL18, IL23R, IL6R, and NFKB2. These genes are involved in the immune/defense response against infection.

We noted that GO term Mitochondrial translation was called over‐represented by DAVID in GX6B(48hpi/0 hpi), but did not form a cluster. Interestingly, this and several other mitochondria‐related terms were called as enriched by GSEA in both GX6B and GX6BsR, and to a moderate extent also in GX6Bs with a smaller set of GO terms. These enriched terms are shown in two functionally related groups of Mitochondria organization and translation, and Mitochondrial ETC (electron transfer chain) and OxPho (oxidative phosphorylation) in Figure 4. In a transcriptomic study of GX2, an identically constructed cell line but different clones as the parental cell of GX6B, it was similarly found that mitochondrial biogenesis and respiration GO terms were called enriched by GSEA but not by DAVID.^25^ The log_2_ fold change of all genes from GSEA identified enriched GO terms in these two functional groups, with DEGs highlighted, were plotted in Figures S7 and S8. Most transcripts were down‐regulated at 48 hpi, although many did not meet |log_2_FC| > 1 to be called differentially expressed. Many were related to mitochondrial translation (e.g., MRPL1/40/48, (mitochondrial ribosomal proteins)) and components of Complex I, III, and IV of the electron transfer chain (ETC) (e.g., NDUFC2/NDUFS2, UQCRB/UQCR10, COA3/COX16). The change in transcript levels on those mitochondrial genes followed a very similar trend as those in Groups I and II; all three 48 hpi/0 hpi comparisons follow the same trend of downregulation, but the extent of downward expression at 48 hpi was more prominent in GX6B and GX6BsR than in GX6Bs.

The downregulation of these genes related to mitochondrial translation and oxidative phosphorylation was possibly indicative of the general downward tuning of translational activities and energy generation in the late stage of virus production. The downregulation of mitochondrial biogenesis may be associated with host antiviral response.^39^

### 3.6 Medium affects rAAV productivity in suspension culture

The adaptation of GX6B to become suspension‐grown GX6Bs consistently showed a reduction in viral gene expression and virus productivity and elicited cellular transcriptome changes. The reversal of GX6Bs to adherent growth reverted many of these changes, making GX6BsR more similar to GX6B than GX6Bs in many aspects. The restoration of the cultivation conditions involved both cell adhesion and using a different medium. In an exploratory study, we tested restoring the chemical environment but keeping GX6Bs in suspension.

**FIGURE 7:**
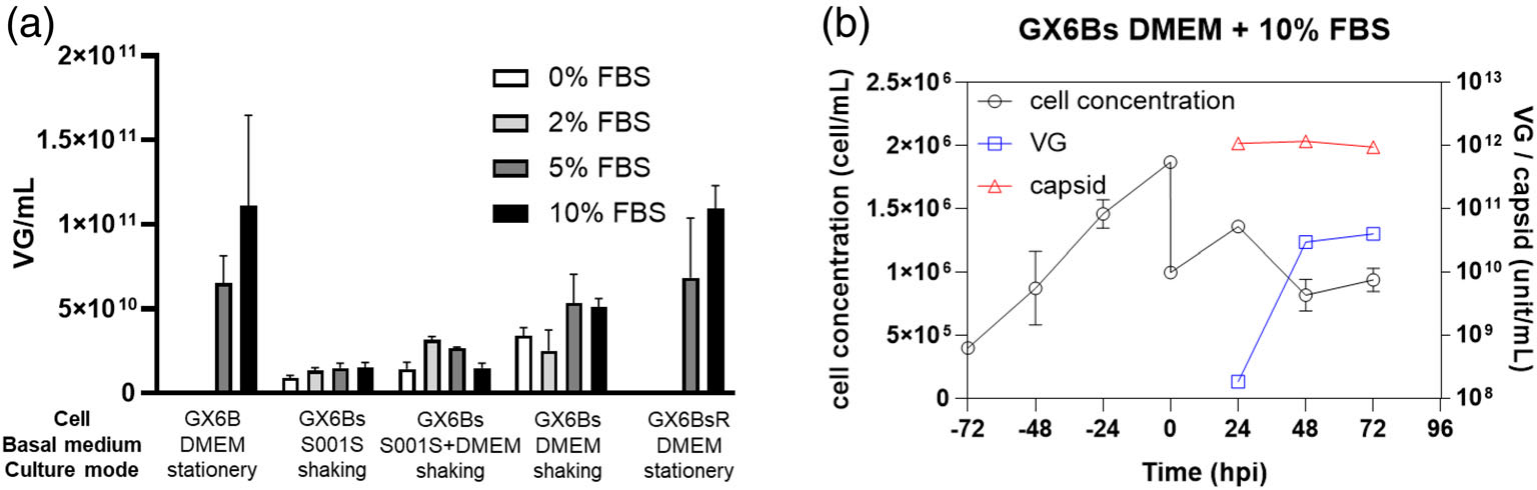
The rAAV productivity and the cell growth of GX6Bs in different induction media. (a) The VG titer of GX6Bs induced in S001S medium, DMEM, or S001S and DMEM (1:1 ratio) with different FBS addition (0%, 2%, 5%, and 10%). The GX6B and re‐adherent GX6BsR induced in DMEM with 5% or 10% FBS addition were used as references. (b) The cell growth and the viral component titer of GX6Bs induced in DMEM with 10% FBS addition during the culturing process; VG (blue) and capsid (red) were measured. The culture conditions are in Supplementary Materials and Methods (Data S1). The error bar stands for SD, *n* = 3.

GX6Bs growing in the mid‐exponential phase in S001S serum‐free medium were transferred to 6‐well plates, cultured in S001S or DMEM as basal medium with different concentrations of FBS, and induced for rAAV production with shaking. The shaking kept cells in suspension. For comparison, two cultures with GX6B and GX6Bs cells growing under adherent conditions were also included. In S001S medium supplementation with up to 10% of FBS, the titer was no more than 8 × 10^3^ VG/mL, less than 1/10 of the adherent culture of GX6B (Figure 7a). Replacing half of S001S medium with DMEM increased VG titer; complete replacement with DMEM improved the titer even further, restoring it to nearly half of the titer of GX6Bs cultivated under adherent conditions (GX6BsR).

The suspension growth medium S001S appears to lack some constituent(s) or properties of DMEM that would boost productivity. For example, the calcium level in media for adapting cells to suspension growth is typically rather low. Hence, it is possible that the high calcium level in DMEM contributed to the increased productivity. It is also plausible that a constituent of S001S had a retarding effect on productivity. Suspension culture media may contain dextran sulfate or heparan to modulate intercellular interactions and reduce cell clumping. One may speculate that reducing the concentration of such agents by mixing with DMEM played a role in virus productivity. The composition of the commercial medium S001S is not available. The root cause of increased productivity with DMEM awaits a systematic study.

It was noted that suspension cells in different culture conditions were also morphologically different. In DMEM medium without FBS, cells aggregated severely (Figure S3D). With 5% FBS, some clumps of 10–20 cells were seen at 48 hpi. With 10% FBS, the aggregate size increased substantially (Figure S3D–G). It is plausible that cell–cell interactions involved in clumping and aggregation facilitate cytoskeletal reorganization and contribute to increased productivity.

The time profile of the suspension culture of GX6Bs in DMEM with 10% FBS is shown in Figure 7b. The VG titer was still lower than that seen in re‐adherent conditions (GX6BsR). However, the culture was performed under routine culture conditions without any improvement. Through process enhancement such as employing fed‐batch or perfusion mode, current industrial suspension cultures can reach more than ten times higher cell density.^40^ Even though a suspension culture has a somewhat lower specific rAAV productivity than its adherent counterpart, its higher cell concentration can more than make up for it. One can envision a process strategy, first expanding the production cell in suspension culture without induction. As serial expansion reaches the production scale, the medium is changed to one that favors adherent growth, but cells would be kept in suspension because of the use of a stirred tank reactor, and the culture is induced for rAAV production. Such a process will be scalable. Since cells are not induced during the expansion stage, the culture is not subjected to selection for cells with lower productivity. Thus, the high productivity characteristics of the cell line can be stable.

Given that a process using synthetic cell lines for rAAV production will likely demand a high cell viability at the time of induction to ensure a high productivity, a perfusion culture with a low metabolite accumulation can be an attractive process option.^41^, ^42^ Perfusion culture has long been used in the production of therapeutic protein biologics as well as viruses including rAAV.^20^, ^40^, ^42^, ^43^, ^44^, ^45^, ^46^ Intensive efforts in process enhancement in the past decade have transformed perfusion cultures to be highly efficient biomanufacturing vehicles for protein biologics. To transform a synthetic stable cell line‐based suspension culture into a highly productive biomanufacturing vehicle, further process enhancement is needed. Note that when GX6Bs adapted to suspension growth, it also adapted to serum‐free growth, switching to DMEM with 10% FBS to boost productivity while also introducing complex components that needed to be removed from the product. With some process optimization, including possibly adopting perfusion and developing the serum‐free medium, the rAAV production using a synthetic cell line can become a scalable suspension culture process.

## 4 CONCLUSIONS

In this work, we demonstrated the suspension adaptation of the rAAV‐producing cell line. The suspension cell was feasible to return to adherent growth in a short time. Upon induction, the rAAV productivity decreased in the suspension culture but was restored in the suspension re‐adherent culture. The transcriptomic and targeted proteomic results showed the behavior of the re‐adherent cells was much more similar to the parental adherent cells compared to the suspension cells. The response after induction in suspension cells was more subdued, while the adherent and re‐adherent cells had major changes in transcriptomic analysis. We also demonstrated the productivity improvement in suspension cells by changing culture medium. The feasibility of the suspension producer cell line for rAAV production to meet industrial needs was demonstrated.

## AUTHOR CONTRIBUTIONS

Han‐Jung Kuo, Yu‐Chieh Lin, Min Lu, Qi Zhang, and Wei‐Shou Hu designed the experimental plan. Han‐Jung Kuo, Yu‐Chieh Lin, and Carissa Rungkittikhun performed all experiments and analyzed the relevant data. Prahalad Srinivasan analyzed the RNA‐seq data. All authors contributed to the revision and final review of the manuscript. Wei‐Shou Hu supervised the study.

## CONFLICT OF INTEREST STATEMENT

The authors declare no conflict of interest.

## Supporting information


**Data S1.** Supporting Information.

## Data Availability

The data that support the findings of this study are available from the corresponding author upon reasonable request.
