# Supplementary material for: Transcriptomic functional characterization of recombinant adeno‐associated virus producing cell line adapted to suspension‐growth
Source: Biotechnol Prog. 2025 May 21;41(5):e70042. doi: 10.1002/btpr.70042 (PMC12531931; doi:10.1002/btpr.70042)
Supplement: Supplementary file 1 — Data S1. Supporting Information. [file BTPR-41-e70042-s001.pdf]

Supporting Information

Transcriptomic Functional Characterization of Recombinant Adeno-Associated Virus Producing Cell Line Adapted to Suspension-Growth

Han-Jung Kuo, Prahalad Srinivasan, Yu-Chieh Lin, Min Lu, Carissa Rungkittikhun, Qi Zhang and Wei-Shou Hu*

Department of Chemical Engineering and Materials Science, University of Minnesota, 421 Washington Avenue S.E., Minneapolis, Minnesota 55455-0132

*__CORRESPONDING AUTHOR__
Wei-Shou Hu
Address: 421 Washington Avenue SE, Minneapolis, MN 55455-0132 USA
Email: wshu@umn.edu

Contents

Supplementary Figures 3
Figure S1. 3
Figure S2. 4
Figure S3. 6
Figure S4 7
Figure S5. 8
Figure S6. 9
Figure S7. 10
Figure S8. 11
Supplementary Materials and methods 11
Suspension growth adaptation 11
rAAV production in GX6Bs 12
Assay of rAAV titer 12
Illumina sequencing and differential expression analysis 12
Functional class analysis 13
Targeted quantitative proteomics analysis 13
Supplementary Tables 14
Table S1. 14
Table S2. 16
Table S3. 17
Table S4. 19
Table S5. 21
Table S6. 22
Reference: 23

SUPPLEMENTARY FIGURES

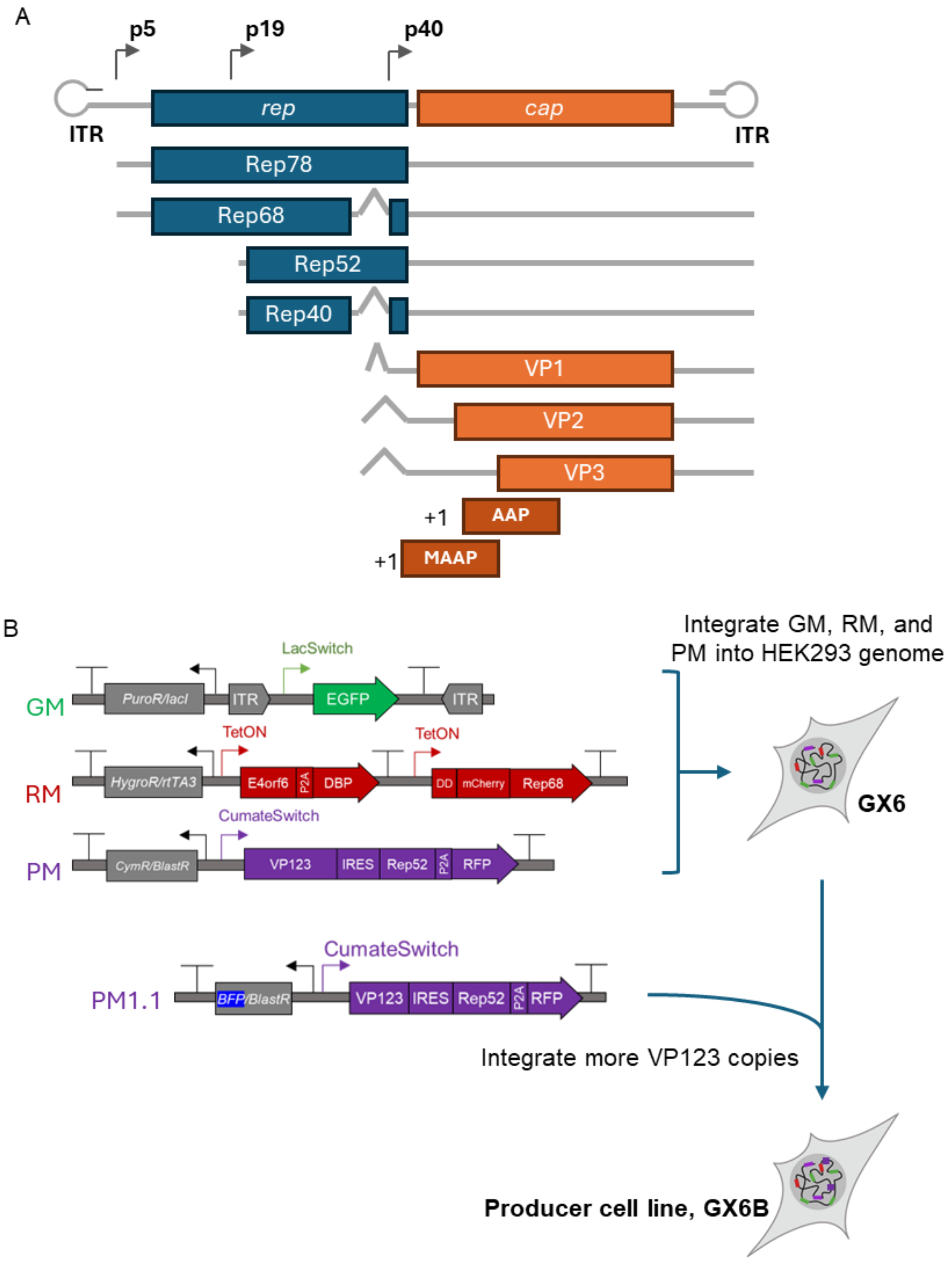

Figure S1. (A) Schematic diagram of AAV genome and the gene products produced by 6 transcripts. (B) Schematics of the construction of GX cell lines and the genetic modules integrated into GX cell genome.

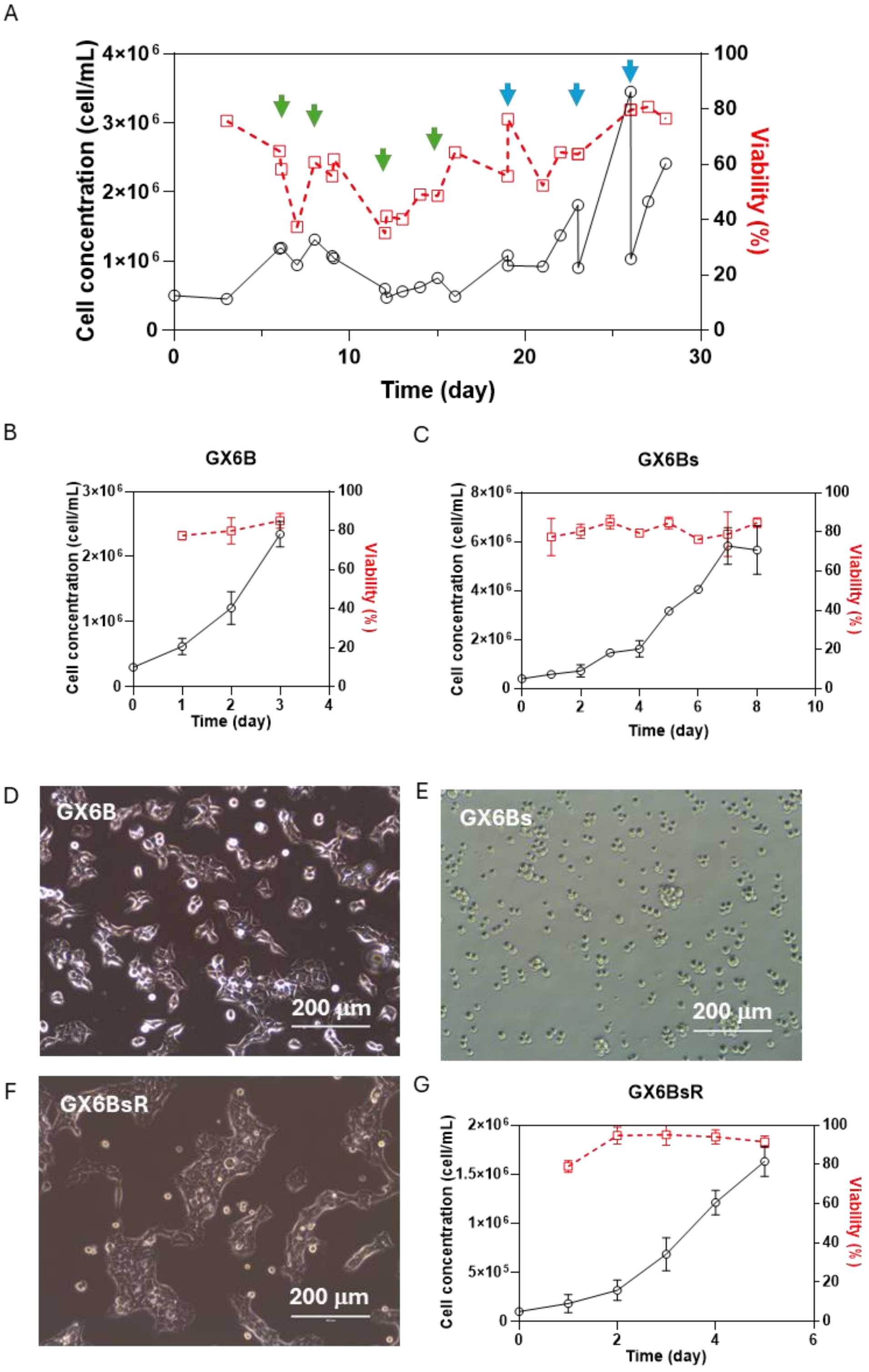

Figure S2. (A) The adaptation process of GX6B to suspension growth in Celer-S001S serum-free medium. The green arrows indicate the time points that 50% of the culture medium was changed to fresh medium. The blue arrows indicate the time points that the culture was diluted two times. (B), (C), (G) The growth profile of

different cultures. (D), (E), (F) The cell morphology of different cell lines.

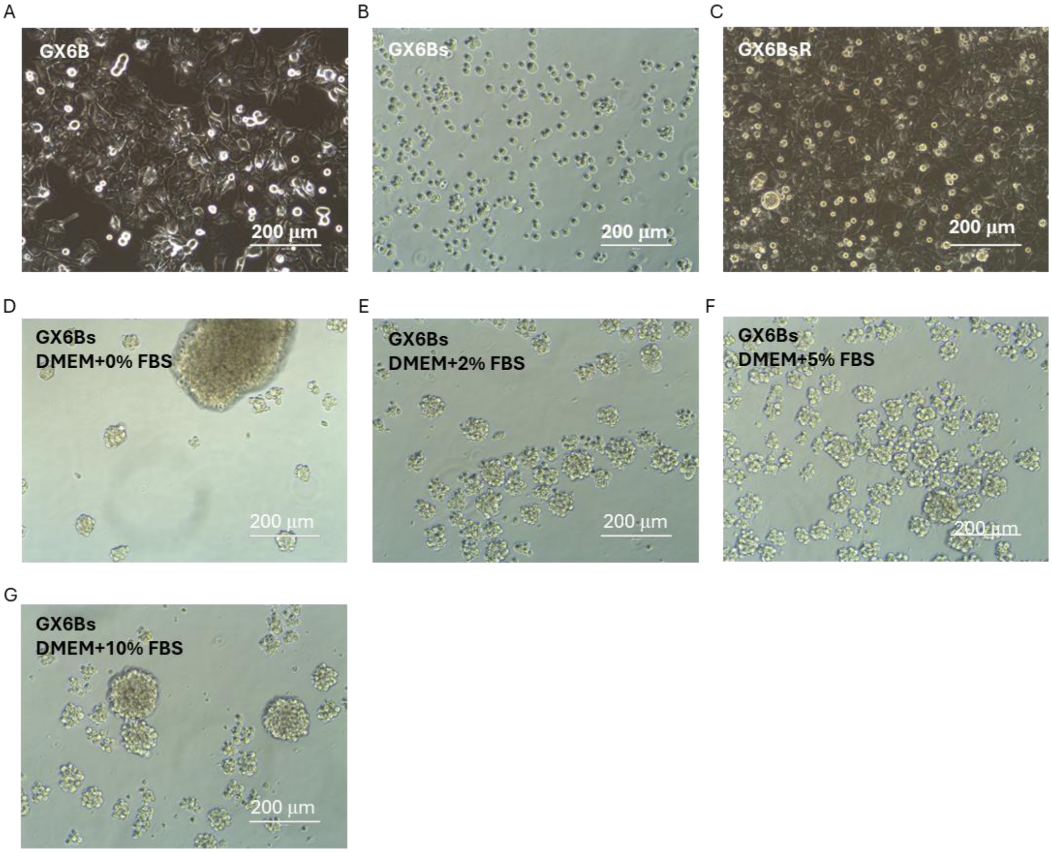

Figure S3. The morphology at 48 hpi of GX6B in DMEM (10% FBS) (A), GX6Bs in S001S medium (no FBS) (B), GX6BsR in DMEM (10% FBS) growing adherently (C), and GX6Bs in DMEM with different concentrations of FBS (D)-(G) growing in suspension.

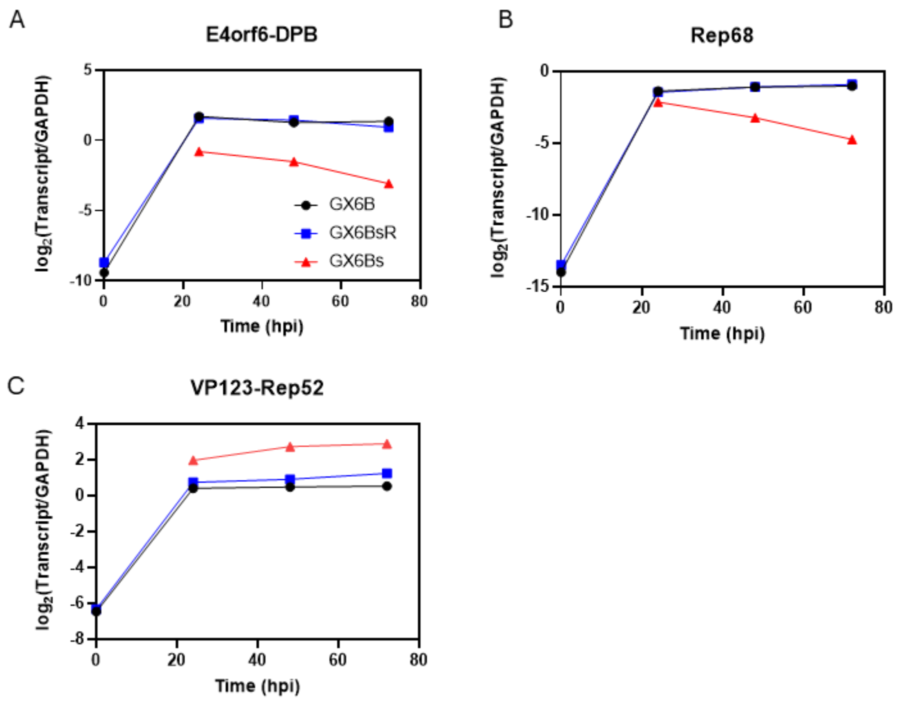

Figure S4. The relative levels of viral transcripts E4orf6-(P2A)-DBP (A), DD-mCherry-Rep68 (B) and VP123-IRES-Rep52-(P2A)-RFP (C) by qRT-PCR. GAPDH was used as the reference gene.

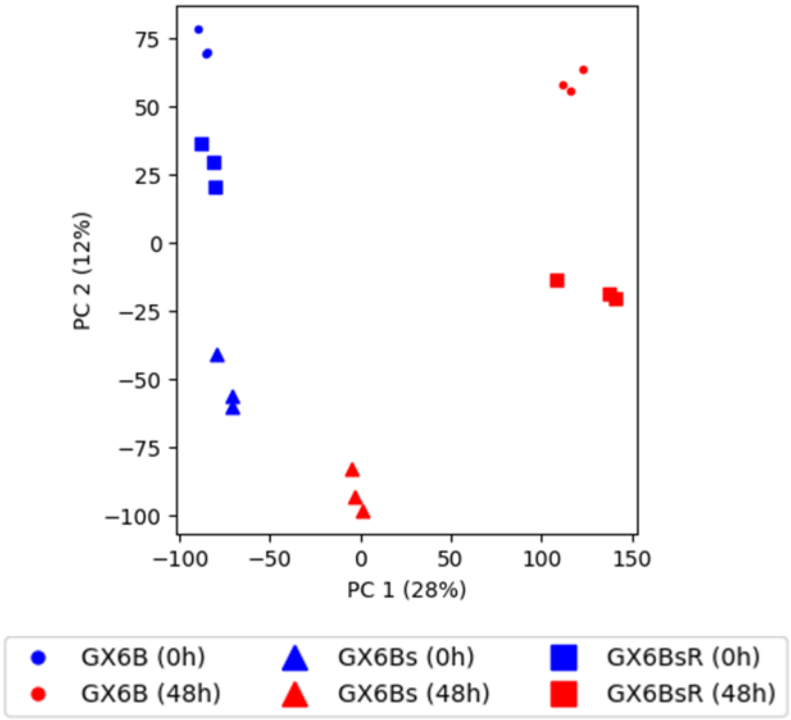

Figure S5. Principal component analysis of transcriptome data (TPM) projections on PC1 vs. PC2 plane. Biological replicates for each sample are closely grouped, indicating consistency of measurements. The induced and uninduced samples were separated by PC1.

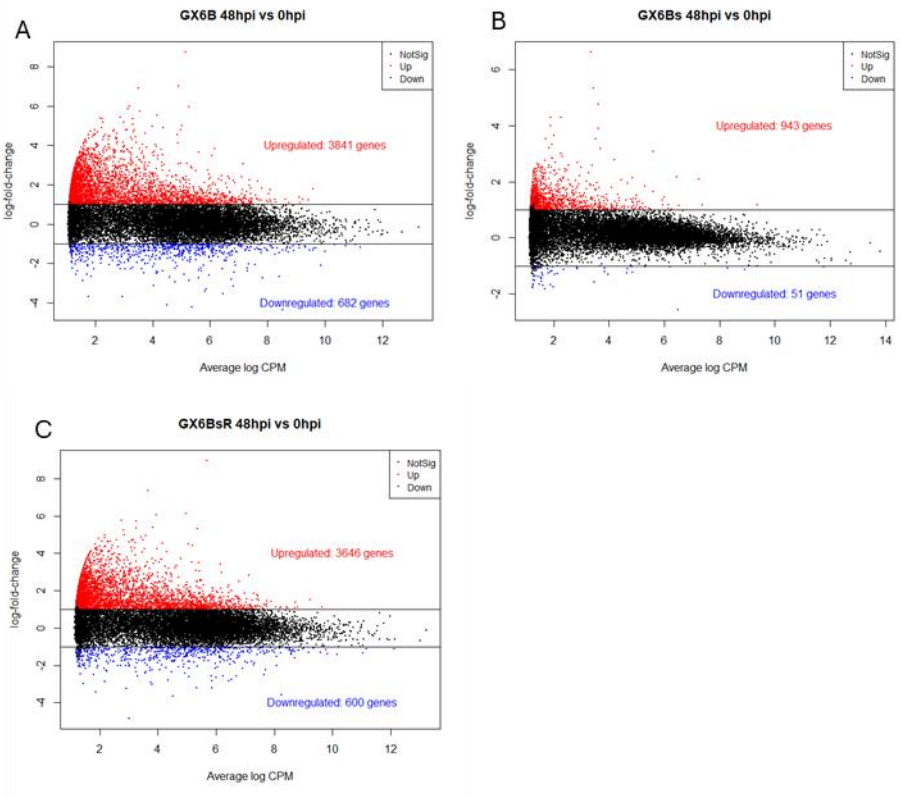

Figure S6. MA plot showing differentially expressed genes across the three cell lines. GX6B (A) and GX6BsR (C) have more differentially expressed genes compared to GX6Bs (B).

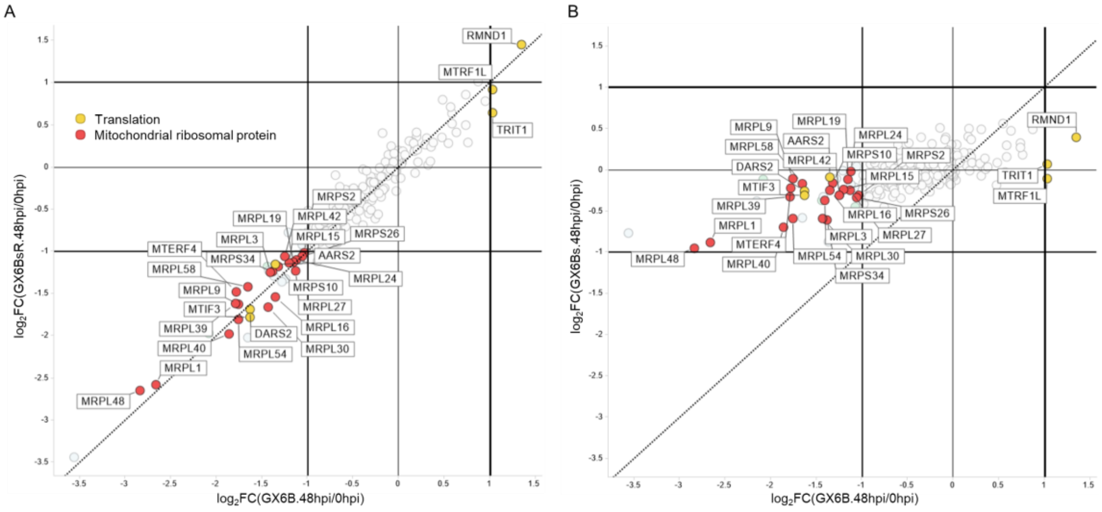

Figure S7. A double differential expression plot of genes in group III, Mitochondria structure and translation. DEGs at 48 hpi in GX6B are labeled. The fold change of the genes after induction in GX6B (log2FC(GX6B.48hpi/0hpi)) was compared to GX6BsR (log2FC(GX6BsR.48hpi/0hpi)) (A) and GX6Bs (log2FC(GX6Bs.48hpi/0hpi)) (B). The dashed line indicates the x=y line. The genes were listed in Table S3 and mainly belonged to 2 groups: translation-related proteins (yellow) and mitochondrial ribosomal proteins (red).

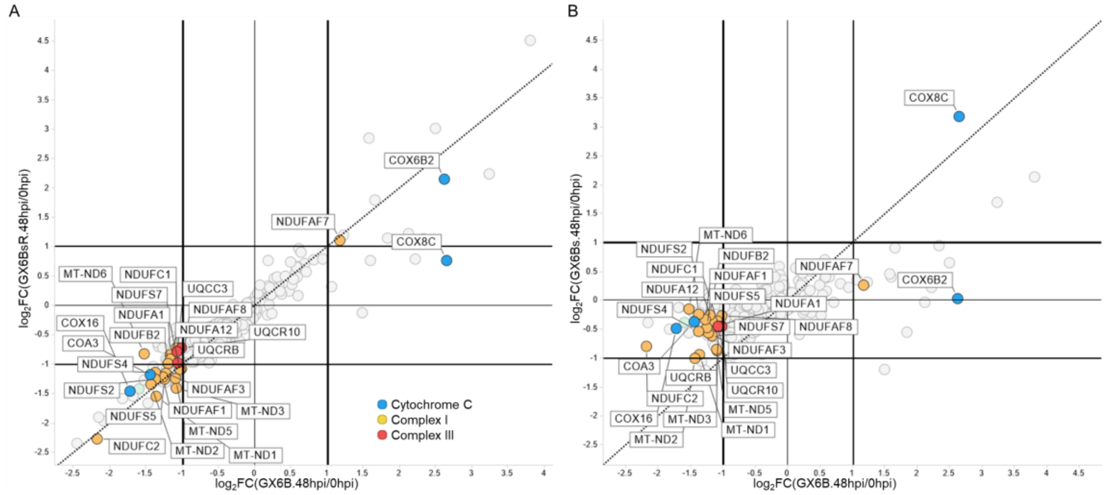

Figure S8. A double differential expression plot of genes in group IV, Mitochondrial electron transport chain and oxidative phosphorylation. DEGs at 48 hpi in GX6B are labeled. The fold change of the genes after induction in GX6B (log2FC(GX6B.48hpi/0hpi)) was compared to GX6BsR (log2FC(GX6BsR.48hpi/0hpi)) (A) and GX6Bs (log2FC(GX6Bs.48hpi/0hpi)) (B). The dashed line indicates the x=y line. The genes were listed in Table S4 and were grouped into 3 groups: cytochrome C (blue), respiratory complex I (yellow), and respiratory complex III (red).

SUPPLEMENTARY MATERIALS AND METHODS

Suspension growth adaptation

The adaptation process was based on the manufacturer's instructions. The GX6B adherent cells were harvested and resuspended in Celer-S001S medium to 5×10^5 cell/mL. The cell culture (30 mL) was placed in a 150 mL shake flask. The cells were grown at 37°C in a humidified 5% CO_2 air atmosphere with 100 rpm shaking. The viable cell density (VCD) was measured every 2 days. If the VCD was below 2×10^6 cell/mL, half of the cells were spun down, resuspended in fresh medium, and combined with the other half. This medium refreshing process was repeated every 7 days. If the VCD reached 2×10^6 cell/mL, the cells were diluted to 1×10^6 cell/mL and split into two flasks. The culture expansion process was repeated until the

viability returned to 80-90%.

rAAV production in GX6Bs

The GX6Bs cells were seeded at 4×10^5 cells/mL in a shake flask with S001S medium and rotating at 100 rpm in an orbital shaker for three days before cells were harvested. S001S, DMEM, and the mixture of S001S and DMEM (1:1 ratio) with 0, 2, 5, or 10% FBS and 10 μg/mL doxycycline and 90 μg/mL cumate were used to induce rAAV production. The cells were harvested and resuspended in the induction medium at 1×10^6 cells/mL. The cells were induced in 6-well plates with shaking for 72 hours.

Assay of rAAV titer

Benzonase treatment of crude cell lysate for rAAV isolation, the quantification of encapsidated vector genomes (VG) and total intracellular virus genomes (TG) by qPCR, the titration of capsids by ELISA were previously described [1, 2]. In the quantification of TG, GPR15 was used as the reference to calculate the copy number. The copies of VG were calculated based on the standard curve generated through a series of serial dilutions of the plasmid standard. The primers used in this study were listed in Table S5.

Illumina sequencing and differential expression analysis

RNA was extracted using the RNeasy Mini kit (Qiagen). Illumina TruSeq Stranded mRNA library preparation was performed by the University of Minnesota Genomics Center. Each library was sequenced as paired-end 150 bp reads on a NovaSeq 6000 instrument at a target depth of 20 M reads. Read mapping, counting, and TPM (transcript per million) calculation were previously described [1]. Differentially expressed genes (DEG) were identified using a quasi-likelihood negative binomial generalized log-linear model in the EdgeR package in R [3]. Genes with a CPM (count per million) smaller than 3 in all samples were excluded from further analysis. Transcripts were considered as DEGs if the false discovery rate (FDR) was below 5% and the log2 fold change (FC) was greater than 1 or less than -1. Only genes with CPM measurements above 2 were considered for DEG analysis. TMM normalization was applied to scale the raw library sizes. PCA was performed based on TPM data. Any TPM measurements below 0.1 were filtered out. TPM was then standardized before performing PCA.

Functional class analysis

Both GSEA and ORA (using DAVID) were performed to identify enriched functional gene sets using the same GO terms of Biological Process. For Gene Set Enrichment Analysis (GSEA) [4, 5], log_2(CPM) data was fed to the GSEA algorithm. Phenotype permutations were used with 1000 permutations. The minimum and maximum gene set sizes considered for analysis were 15 and 500, respectively. Gene sets were considered enriched when the normalized enrichment score was above 1, the p-value was below 0.05, and FDR was below 25%. Overrepresentation analysis (ORA) and functional annotation clustering were carried out using DAVID bioinformatics resources [6, 7]. The DEGs (both up- and down-regulated) were fed to the DAVID algorithm. Gene sets were considered enriched when the p-value was below 0.05, and the FDR was below 25%.

Targeted quantitative proteomics analysis

Protein sample preparation, parameters used for acquiring parallel reaction monitoring (PRM)-based targeted mass spectrometry data, and raw data processing on Skyline were based on the developed protocols [8] and described in detail previously [1, 9]. The ratio of light to heavy peptides was calculated from the peak areas, and the ratios were then used to determine the endogenous protein concentrations. Human beta-actin proteins encoded by ACTB (UniProt accession: P60709) were quantified and used as an internal control. The sequences of the heavy isotope-labeled peptides used in this study were listed in Table S6. Viral protein copies per cell were calculated based on the assumption that the total protein of a HEK293 cell is 360 pg.

SUPPLEMENTARY TABLES

Table S1. The representative DEGs in group I, nucleosome organization. The logFC represents

the log_2FC(48hpi/0hpi). The genes were grouped based on their function.

Gene name	Gene description	logFC GX6B	logFC GX6Bs	logFC GX6BsR	TPM GX6B 0h	TPM GX6B 48h	Subgroup
ANP32B	acidic nuclear phosphoprotein 32 family member B(ANP32B)	-1.29	-0.28	-1.24	190.14	96.84	DNA/chromatin-binding
CHD5	chromodomain helicase DNA binding protein 5(CHD5)	2.91	1.69	2.65	0.23	1.61	DNA/chromatin-binding
DMC1	DNA meiotic recombinase 1(DMC1)	2.07	0.58	2.13	0.95	4.43	DNA/chromatin-binding
FOXA3	forkhead box A3(FOXA3)	2.48	1.95	2.43	0.29	1.98	DNA/chromatin-binding
GTF2H5	general transcription factor IIH subunit 5(GTF2H5)	-1.60	-0.45	-1.52	19.65	7.28	DNA/chromatin-binding
HMGB1	high mobility group box 1(HMGB1)	-1.67	-0.49	-1.55	500.50	182.89	DNA/chromatin-binding
ITGB3BP	integrin subunit beta 3 binding protein(ITGB3BP)	-1.10	-0.24	-0.82	60.19	26.45	DNA/chromatin-binding
MED20	mediator complex subunit 20(MED20)	-1.30	-0.20	-1.20	29.90	14.70	DNA/chromatin-binding
MED23	mediator complex subunit 23(MED23)	-1.19	-0.06	-1.15	16.69	8.45	DNA/chromatin-binding
MYC	MYC proto-oncogene, bHLH transcription factor(MYC)	-3.79	-0.50	-2.82	140.90	14.16	DNA/chromatin-binding
NFE2	nuclear factor, erythroid 2(NFE2)	3.55	1.21	2.55	0.15	1.29	DNA/chromatin-binding
PARP1	poly(ADP-ribose) polymerase 1(PARP1)	-1.14	-0.28	-1.18	350.17	188.51	DNA/chromatin-binding
PARP10	poly(ADP-ribose) polymerase family member 10(PARP10)	2.31	-0.12	1.56	0.57	3.63	DNA/chromatin-binding
PRIMPOL	primase and DNA directed polymerase(PRIMPOL)	-1.10	0.12	-1.05	10.43	5.21	DNA/chromatin-binding
SMARCB1	SWI/SNF related, matrix associated, actin dependent regulator of chromatin, subfamily b, member 1(SMARCB1)	-1.43	-0.19	-1.63	149.06	70.72	DNA/chromatin-binding
VCPIP1	valosin containing protein interacting protein 1(VCPIP1)	1.23	0.52	1.60	4.87	12.92	DNA/chromatin-binding
H1-2	H1.2 linker histone, cluster member(H1-2)	3.27	2.97	3.21	31.51	368.19	histone
H1-4	H1.4 linker histone, cluster member(H1-4)	1.50	1.13	2.13	0.61	1.73	histone
H2AC25	H2A clustered histone 25(H2AC25)	2.59	1.82	2.37	26.50	202.18	histone
H2AC8	H2A clustered histone 8(H2AC8)	4.90	3.34	3.65	0.11	7.55	histone
H2BC11	H2B clustered histone 11(H2BC11)	3.62	2.50	2.80	6.28	85.00	histone
H2BC13	H2B clustered histone 13(H2BC13)	1.79	1.91	2.53	5.05	55.26	histone
H2BC15	H2B clustered histone 15(H2BC15)	4.78	1.93	3.95	1.92	59.08	histone
H2BC17	H2B clustered histone 17(H2BC17)	5.91	2.76	4.69	0.52	35.18	histone
H2BC21	H2B clustered histone 21(H2BC21)	4.37	2.69	3.58	2.83	66.20	histone
H2BC4	H2B clustered histone 4(H2BC4)	2.88	2.28	2.99	3.38	27.75	histone
H2BC6	H2B clustered histone 6(H2BC6)	2.28	1.68	1.88	0.80	2.60	histone
H2BC7	H2B clustered histone 7(H2BC7)	4.02	2.57	2.76	0.22	3.70	histone
H2BC8	H2B clustered histone 8(H2BC8)	3.75	2.29	3.29	0.12	3.81	histone
H2BC9	H2B clustered histone 9(H2BC9)	2.22	-0.04	1.54	0.93	5.69	histone

Gene name	Gene description	logFC GX6B	logFC GX6Bs	logFC GX6BsR	TPM GX6B 0h	TPM GX6B 48h	Subgroup
H3C1	H3 clustered histone 1(H3C1)	2.13	1.65	1.43	0.32	1.93	histone
H3C10	H3 clustered histone 10(H3C10)	3.54	2.57	3.20	3.92	54.65	histone
H3C12	H3 clustered histone 12(H3C12)	2.79	2.91	5.24	1.08	11.88	histone
H3C13	H3 clustered histone 13(H3C13)	3.74	0.45	1.15	0.00	1.77	histone
H3C14	H3 clustered histone 14(H3C14)	4.55	2.29	7.66	0.15	4.05	histone
H3C15	H3 clustered histone 15(H3C15)	7.67	1.82	3.59	0.00	3.05	histone
H3C6	H3 clustered histone 6(H3C6)	3.49	2.18	3.57	2.37	25.98	histone
H3C7	H3 clustered histone 7(H3C7)	1.79	0.74	-0.54	1.02	2.96	histone
H4C11	H4 clustered histone 11(H4C11)	2.15	0.81	2.12	0.19	1.03	histone
H4C12	H4 clustered histone 12(H4C12)	7.65	2.14	2.13	0.00	1.05	histone
H4C14	H4 clustered histone 14(H4C14)	3.30	2.35	2.37	2.74	39.17	histone
H4C15	H4 clustered histone 15(H4C15)	3.42	3.75	2.18	1.08	12.30	histone
H4C16	H4 histone 16(H4C16)	1.94	1.35	1.34	0.53	4.67	histone
H4C4	H4 clustered histone 4(H4C4)	4.33	2.29	3.92	0.29	7.93	histone
H4C5	H4 clustered histone 5(H4C5)	6.38	3.00	6.18	0.23	25.14	histone
H4C8	H4 clustered histone 8(H4C8)	5.82	3.86	5.21	1.78	126.84	histone
H4C9	H4 clustered histone 9(H4C9)	4.05	1.31	2.65	0.07	3.34	histone
APLF	aprataxin and PNKP like factor(APLF)	1.22	0.39	0.69	0.50	1.18	histone modification
HDAC5	histone deacetylase 5(HDAC5)	1.29	0.50	1.42	14.80	42.97	histone modification
HDAC8	histone deacetylase 8(HDAC8)	-1.49	-0.38	-1.28	18.86	8.52	histone modification
KDM5B	lysine demethylase 5B(KDM5B)	2.00	0.69	1.56	13.13	60.06	histone modification
KDM6B	lysine demethylase 6B(KDM6B)	2.20	0.97	1.65	5.56	35.36	histone modification
MBTD1	mbt domain containing 1(MBTD1)	1.40	0.62	1.12	13.90	37.62	histone modification
SGF29	SAGA complex associated factor 29(SGF29)	-1.35	-0.42	-1.74	12.90	6.29	histone modification

Table S2. The representative DEGs in group I, immune/defense response. The logFC represents the log_2FC(48hpi/0hpi).

Gene name	Gene description	logFC GX6B	logFC GX6Bs	logFC GX6BsR	TPM GX6B 0h	TPM GX6B 48h	Subgroup
CHGA	chromogranin A(CHGA)	2.14	1.62	2.28	1.06	4.55	antimicrobial
LEAP2	liver enriched antimicrobial peptide 2(LEAP2)	1.03	1.07	1.57	1.46	3.83	antimicrobial
LYG1	lysozyme g1(LYG1)	2.28	0.33	1.76	0.86	3.76	antimicrobial
SEMG1	semenogelin 1(SEMG1)	-3.34	-0.38	-2.20	0.34	0.03	antimicrobial
SLPI	secretory leukocyte peptidase inhibitor(SLPI)	2.62	0.50	4.51	0.47	4.14	antimicrobial
C2	complement C2(C2)	3.38	1.34	3.74	0.63	8.56	complement
C3	complement C3(C3)	4.84	2.44	2.78	0.35	3.65	complement
C5AR1	complement C5a receptor 1(C5AR1)	3.30	1.67	4.23	0.26	4.88	complement
CFB	complement factor B(CFB)	3.17	4.66	3.95	0.19	3.13	complement
CXCL11	C-X-C motif chemokine ligand 11(CXCL11)	6.28	1.33	2.89	0.00	0.59	cytokine
F2	coagulation factor II, thrombin(F2)	2.94	4.56	3.19	0.00	0.38	cytokine
GBP2	guanylate binding protein 2(GBP2)	4.03	-0.02	2.72	0.08	0.63	cytokine
IFI44	interferon induced protein 44(IFI44)	1.51	-0.28	1.41	0.50	1.18	cytokine
IL18	interleukin 18(IL18)	6.72	1.05	7.17	0.00	1.55	cytokine
IL1A	interleukin 1 alpha(IL1A)	4.73	2.85	5.02	0.02	0.67	cytokine
IL22RA1	interleukin 22 receptor subunit alpha 1(IL22RA1)	3.65	-0.21	5.17	0.00	0.26	cytokine
IL23R	interleukin 23 receptor(IL23R)	8.95	0.00	7.26	0.00	1.39	cytokine
IL6R	interleukin 6 receptor(IL6R)	1.61	0.91	2.29	1.23	4.20	cytokine
IL7R	interleukin 7 receptor(IL7R)	3.54	1.62	3.28	0.00	0.42	cytokine
IRAK3	interleukin 1 receptor associated kinase 3(IRAK3)	1.35	0.69	1.94	0.21	0.75	cytokine
IRF5	interferon regulatory factor 5(IRF5)	1.17	0.14	0.77	1.68	4.31	cytokine
IRF8	interferon regulatory factor 8(IRF8)	1.88	0.38	2.22	0.72	2.01	cytokine
LTA	lymphotoxin alpha(LTA)	3.72	3.62	4.82	0.19	4.07	cytokine
NFKB2	nuclear factor kappa B subunit 2(NFKB2)	1.63	0.45	1.48	6.05	24.96	cytokine
NFKBIL1	NFKB inhibitor like 1(NFKBIL1)	1.10	0.37	0.99	26.74	68.49	cytokine
NLRP3	NLR family pyrin domain containing 3(NLRP3)	2.58	0.84	1.18	0.05	0.42	cytokine
SSC5D	scavenger receptor cysteine rich family member with 5 domains(SSC5D)	2.91	0.08	2.82	0.10	0.95	cytokine
TNFSF4	TNF superfamily member 4(TNFSF4)	2.46	1.80	2.44	0.40	2.51	cytokine
H2BC11	H2B clustered histone 11(H2BC11)	3.62	2.50	2.80	6.28	85.00	H2B
H2BC12	H2B clustered histone 12(H2BC12)	2.80	2.04	2.61	115.76	1001.71	H2B
H2BC12L	H2B clustered histone 12 like(H2BC12L)	2.98	1.59	2.34	9.29	94.19	H2B
H2BC21	H2B clustered histone 21(H2BC21)	4.37	2.69	3.58	2.83	66.20	H2B
H2BC4	H2B clustered histone 4(H2BC4)	2.88	2.28	2.99	3.38	27.75	H2B
H2BC6	H2B clustered histone 6(H2BC6)	2.28	1.68	1.88	0.80	2.60	H2B
H2BC7	H2B clustered histone 7(H2BC7)	4.02	2.57	2.76	0.22	3.70	H2B
H2BC8	H2B clustered histone 8(H2BC8)	3.75	2.29	3.29	0.12	3.81	H2B

Table S3. The representative DEGs in group III, mitochondria structure and translation. The logFC represents the log_2FC(48hpi/0hpi).

Gene name	Gene description	logFC GX6B	logFC GX6Bs	logFC GX6BsR	TPM GX6B 0h	TPM GX6B 48h	Subgroup
UQCC3	ubiquinol-cytochrome c reductase complex assembly factor 3(UQCC3)	-1.07	-0.45	-0.79	20.74	11.27	complexIII
COA3	cytochrome c oxidase assembly factor 3(COA3)	-1.44	-0.37	-1.18	210.63	95.56	cytochrome c
OMA1	OMA1 zinc metallopeptidase(OMA1)	-2.08	-0.12	-1.97	17.83	3.87	cytochrome c
MRPL1	mitochondrial ribosomal protein L1(MRPL1)	-2.66	-0.88	-2.58	29.67	7.18	MRP
MRPL15	mitochondrial ribosomal protein L15(MRPL15)	-1.2	-0.24	-1.14	154.09	76.13	MRP
MRPL16	mitochondrial ribosomal protein L16(MRPL16)	-1.35	-0.25	-1.54	74.85	34.97	MRP
MRPL19	mitochondrial ribosomal protein L19(MRPL19)	-1.15	-0.12	-1.13	56.17	30.26	MRP
MRPL24	mitochondrial ribosomal protein L24(MRPL24)	-1.12	-0.02	-1.1	148.55	84.53	MRP
MRPL27	mitochondrial ribosomal protein L27(MRPL27)	-1.13	-0.25	-1.23	188.08	102.13	MRP
MRPL3	mitochondrial ribosomal protein L3(MRPL3)	-1.41	-0.37	-1.25	140.09	59.23	MRP
MRPL30	mitochondrial ribosomal protein L30(MRPL30)	-1.43	-0.59	-1.66	42.81	18.19	MRP
MRPL39	mitochondrial ribosomal protein L39(MRPL39)	-1.79	-0.33	-1.62	62.97	22.01	MRP
MRPL40	mitochondrial ribosomal protein L40(MRPL40)	-1.86	-0.7	-1.98	95.62	30.87	MRP
MRPL42	mitochondrial ribosomal protein L42(MRPL42)	-1.25	-0.31	-1.06	161.16	84.25	MRP
MRPL48	mitochondrial ribosomal protein L48(MRPL48)	-2.84	-0.95	-2.65	64.32	9.32	MRP
MRPL54	mitochondrial ribosomal protein L54(MRPL54)	-1.76	-0.59	-1.81	55.87	18.76	MRP
MRPL58	mitochondrial ribosomal protein L58(MRPL58)	-1.65	-0.17	-1.42	70.76	24.08	MRP
MRPL9	mitochondrial ribosomal protein L9(MRPL9)	-1.76	-0.11	-1.63	126.92	37.4	MRP
MRPS10	mitochondrial ribosomal protein S10(MRPS10)	-1.32	-0.16	-1.18	52.15	23.66	MRP
MRPS2	mitochondrial ribosomal protein S2(MRPS2)	-1.03	-0.31	-1.02	138.01	85.58	MRP
MRPS26	mitochondrial ribosomal protein S26(MRPS26)	-1.06	-0.34	-1.06	87.38	50.82	MRP
MRPS34	mitochondrial ribosomal protein S34(MRPS34)	-1.38	-0.61	-1.24	218.63	101.3	MRP
MTERF4	mitochondrial transcription termination factor 4(MTERF4)	-1.78	-0.22	-1.48	33.03	11.93	MRP
AARS2	alanyl-tRNA synthetase 2, mitochondrial(AARS2)	-1.35	-0.09	-1.15	19.98	9.53	translation
DARS2	aspartyl-tRNA synthetase 2, mitochondrial(DARS2)	-1.63	-0.26	-1.69	60.32	22.84	translation
MTIF3	mitochondrial translational initiation factor 3(MTIF3)	-1.63	-0.31	-1.78	31.33	13.11	translation
MTRF1L	mitochondrial translation release factor 1 like(MTRF1L)	1.03	-0.11	0.92	38.33	88.7	translation
RMND1	required for meiotic nuclear division 1 homolog(RMND1)	1.35	0.39	1.45	29.23	82.22	translation
TRIT1	tRNA isopentenyltransferase 1(TRIT1)	1.03	0.07	0.64	21.14	49.95	translation
AGK	acylglycerol kinase(AGK)	-3.56	-0.77	-3.44	46.81	5.25	others
BAX	BCL2 associated X, apoptosis regulator(BAX)	-1.65	-0.58	-2.02	242.85	95.5	others
CDK5RAP1	CDK5 regulatory subunit associated protein 1(CDK5RAP1)	-1.05	-0.28	-1.02	30.93	15.97	others
FASTK	Fas activated serine/threonine kinase(FASTK)	-1.08	-0.21	-1.1	132.66	79.16	others
GADD45GIP1	GADD45G interacting protein 1(GADD45GIP1)	-1.42	-0.33	-1.23	58.84	26.87	others

Gene name	Gene description	logFC GX6B	logFC GX6Bs	logFC GX6BsR	TPM GX6B 0h	TPM GX6B 48h	Subgroup
PRKAA1	protein kinase AMP-activated catalytic subunit alpha 1(PRKAA1)	-1.06	0.04	-0.92	34.47	21.77	others
RCC1L	RCC1 like(RCC1L)	-1.24	-0.3	-1.33	33.37	17.21	others
SARS2	seryl-tRNA synthetase 2, mitochondrial(SARS2)	-1.21	-0.01	-0.78	122.38	61.32	others
TACO1	translational activator of cytochrome c oxidase I(TACO1)	-2.15	-0.38	-1.9	74.92	20.3	others
TIMM10B	translocase of inner mitochondrial membrane 10B(TIMM10B)	-1.12	-0.18	-1.04	29.6	16.47	others
TOMM20	translocase of outer mitochondrial membrane 20(TOMM20)	-1.37	-0.87	-1.29	367.44	172.73	others
TRUB2	TruB pseudouridine synthase family member 2(TRUB2)	-1.28	-0.11	-1.36	27.08	11.35	others

Table S4. The representative DEGs in group IV, mitochondrial ETC and oxidative phosphorylation. The logFC represents the log_2FC(48hpi/0hpi).

Gene name	Gene description	logFC GX6B	logFC GX6Bs	logFC GX6BsR	TPM GX6B 0h	TPM GX6B 48h	Subgroup
ATP5MC2	ATP synthase membrane subunit c locus 2(ATP5MC2)	-1.43	-0.38	-1.59	851.29	320.17	ATP synthase
ATP5ME	ATP synthase membrane subunit e(ATP5ME)	-0.80	-0.45	-1.08	742.81	426.13	ATP synthase
MT-ND1	mitochondrially encoded NADH dehydrogenase 1(MT-ND1)	-1.55	-0.94	-1.36	6283.97	2818.51	complexI
MT-ND2	mitochondrially encoded NADH dehydrogenase 2(MT-ND2)	-1.34	-1.00	-1.43	8590.69	3586.42	complexI
MT-ND3	mitochondrially encoded NADH dehydrogenase 3(MT-ND3)	-1.41	-0.87	-1.08	8403.81	4518.62	complexI
MT-ND5	mitochondrially encoded NADH dehydrogenase 5(MT-ND5)	-1.24	-0.85	-1.09	3012.56	1547.97	complexI
MT-ND6	mitochondrially encoded NADH dehydrogenase 6(MT-ND6)	-0.83	-0.15	-1.52	1122.69	442.29	complexI
NDUFA1	NADH:ubiquinone oxidoreductase subunit A1(NDUFA1)	-1.09	-0.42	-1.03	190.73	115.47	complexI
NDUFA12	NADH:ubiquinone oxidoreductase subunit A12(NDUFA12)	-0.92	-0.36	-1.16	204.01	103.83	complexI
NDUFAF1	NADH:ubiquinone oxidoreductase complex assembly factor 1(NDUFAF1)	-1.21	-0.32	-1.27	22.95	10.64	complexI
NDUFAF3	NADH:ubiquinone oxidoreductase complex assembly factor 3(NDUFAF3)	-1.15	-0.56	-1.22	128.21	71.50	complexI
NDUFAF7	NADH:ubiquinone oxidoreductase complex assembly factor 7(NDUFAF7)	1.11	0.27	1.18	9.16	22.10	complexI
NDUFAF8	NADH:ubiquinone oxidoreductase complex assembly factor 8(NDUFAF8)	-1.07	-0.27	-1.01	458.31	255.14	complexI
NDUFB2	NADH:ubiquinone oxidoreductase subunit B2(NDUFB2)	-0.99	-0.26	-1.19	389.85	192.03	complexI
NDUFC1	NADH:ubiquinone oxidoreductase subunit C1(NDUFC1)	-0.72	-0.36	-1.08	173.52	87.11	complexI
NDUFC2	NADH:ubiquinone oxidoreductase subunit C2(NDUFC2)	-2.27	-0.80	-2.18	543.57	134.25	complexI
NDUFS2	NADH:ubiquinone oxidoreductase core subunit S2(NDUFS2)	-1.25	-0.23	-1.37	92.36	41.17	complexI
NDUFS4	NADH:ubiquinone oxidoreductase subunit S4(NDUFS4)	-1.14	-0.53	-1.37	68.79	27.87	complexI
NDUFS5	NADH:ubiquinone oxidoreductase subunit S5(NDUFS5)	-1.26	-0.47	-1.25	521.36	259.41	complexI
NDUFS7	NADH:ubiquinone oxidoreductase core subunit S7(NDUFS7)	-0.84	-0.62	-1.16	98.22	56.38	complexI
UQCC3	ubiquinol-cytochrome c reductase complex assembly factor 3(UQCC3)	-0.79	-0.45	-1.07	20.74	11.27	complexIII
UQCR10	ubiquinol-cytochrome c reductase, complex III subunit X(UQCR10)	-0.72	-0.45	-1.01	263.12	158.66	complexIII
UQCRB	ubiquinol-cytochrome c reductase binding protein(UQCRB)	-0.98	-0.46	-1.06	305.95	177.42	complexIII
COA3	cytochrome c oxidase assembly factor 3(COA3)	-1.18	-0.37	-1.44	210.63	95.56	cyt c
COX16	cytochrome c oxidase assembly factor COX16(COX16)	-1.47	-0.49	-1.72	64.25	22.41	cyt c
COX6B2	cytochrome c oxidase subunit 6B2(COX6B2)	2.15	0.03	2.62	0.15	1.28	cyt c
COX8C	cytochrome c oxidase subunit 8C(COX8C)	0.77	3.18	2.65	0.11	1.55	cyt c
ACTN3	actinin alpha 3(ACTN3)	1.14	0.95	2.34	0.02	0.12	others
ADCY10	adenylate cyclase 10(ADCY10)	2.24	1.70	3.23	0.15	0.74	others

Gene name	Gene description	logFC GX6B	logFC GX6Bs	logFC GX6BsR	TPM GX6B 0h	TPM GX6B 48h	Subgroup
ANTKMT	adenine nucleotide translocase lysine methyltransferase(ANTKMT)	-1.66	-0.18	-1.47	14.95	7.98	others
CBFA2T3	CBFA2/RUNX1 partner transcriptional co-repressor 3(CBFA2T3)	1.22	-0.04	2.12	0.22	0.95	others
CTPS1	CTP synthase 1(CTPS1)	-1.28	-0.22	-1.36	148.37	66.06	others
CYBA	cytochrome b-245 alpha chain(CYBA)	-1.13	-0.74	-1.16	106.19	60.27	others
DMAC1	distal membrane arm assembly component 1(DMAC1)	-1.29	-0.09	-1.28	57.55	30.36	others
DNAJC15	DnaJ heat shock protein family (Hsp40) member C15(DNAJC15)	-2.37	-0.12	-1.67	0.16	0.08	others
DTYMK	deoxythymidylate kinase(DTYMK)	-1.11	-0.10	-1.18	101.25	55.33	others
FXN	frataxin(FXN)	-2.35	-0.21	-2.45	31.64	6.18	others
IDH1	isocitrate dehydrogenase (NADP(+)) 1(IDH1)	0.32	0.35	1.07	45.34	122.25	others
LYRM7	LYR motif containing 7(LYRM7)	-0.91	0.12	-1.22	13.08	7.48	others
ME3	malic enzyme 3(ME3)	3.02	0.65	2.50	0.16	0.54	others
NIPSNAP2	nipsnap homolog 2(NIPSNAP2)	-1.27	-0.56	-1.52	71.05	29.21	others
NME3	NME/NM23 nucleoside diphosphate kinase 3(NME3)	-1.31	-0.60	-1.23	134.76	64.19	others
NME4	NME/NM23 nucleoside diphosphate kinase 4(NME4)	-0.96	-0.38	-1.01	393.89	236.71	others
NME9	NME/NM23 family member 9(NME9)	0.79	0.23	2.22	0.22	1.12	others
NUPR1	nuclear protein 1, transcriptional regulator(NUPR1)	4.51	2.14	3.81	0.90	13.42	others
OGDHL	oxoglutarate dehydrogenase L(OGDHL)	-1.03	-0.32	-1.40	2.31	0.97	others
OMA1	OMA1 zinc metallopeptidase(OMA1)	-1.97	-0.12	-2.08	17.83	3.87	others
PARP1	poly(ADP-ribose) polymerase 1(PARP1)	-1.18	-0.28	-1.14	350.17	188.51	others
PET117	PET117 cytochrome c oxidase chaperone(PET117)	-1.55	-0.12	-1.68	19.51	9.63	others
PPARA	peroxisome proliferator activated receptor alpha(PPARA)	1.20	0.31	1.23	3.77	9.68	others
PPARGC1A	PPARG coactivator 1 alpha(PPARGC1A)	2.84	0.46	1.57	0.73	3.31	others
STMP1	short transmembrane mitochondrial protein 1(STMP1)	-1.06	-0.35	-1.21	67.53	32.38	others
SUCLG2	succinate-CoA ligase GDP-forming subunit beta(SUCLG2)	-1.58	-0.50	-1.82	23.25	7.69	others
TACO1	translational activator of cytochrome c oxidase I(TACO1)	-1.90	-0.38	-2.15	74.92	20.30	others
TRPV4	transient receptor potential cation channel subfamily V member 4(TRPV4)	1.79	0.91	1.66	0.23	0.90	others
TYMS	thymidylate synthetase(TYMS)	-1.37	-0.30	-1.10	468.00	257.37	others
UCKL1	uridine-cytidine kinase 1 like 1(UCKL1)	-1.13	-0.49	-1.04	31.27	18.24	others
UCN	urocortin(UCN)	0.77	0.41	1.60	1.16	2.81	others

Table S5. Primers used in qPCR assay.

Targeted Transcript	Sequence (5’-3’)
GFP	F: TTCAAGGACGACGGCAACTAC
	R: TCGATGCCCTTCAGCTCGAT
Rep68 CDS	F: GGAACCCCTAGTGATGGAGTT
	R: CGGCCTCAGTGAGCGA
DBP CDS	F: CAAGGCCAAGATCGTGAAGA
	R: AGAAGAACATGCCGCAAGA
VP123 CDS	F: GGGTTCTCATCTTTGGGAAG
	R: GGGATTGGTTGTCCTGATTT
GAPDH	F: GAAGGTGAAGGTCGGAGTC
	R: GAAGATGGTGATGGGATTTC
GPR15	F: GGTCCCTGGTGGCCTTAATT
	R: TTGCTGGTAATGGGCACACA

Table S6. List of heavy isotope-labeled peptides used in the targeted proteomics analysis for absolute quantification (AQUA).

Targeted protein	Sequence
ACTB	AGFAGDDAP[R_C13N15]
GFP	FSVSGEGEGDATYG[K_C13N15]
Rep68	DFLTEW[R_C13N15]
Rep68/Rep52	AILGGS[K_C13N15]
DBP	NVSLPVAHSDA[R_C13N15]
E4orf6	EELVIL[R_C13N15]
VP1/VP2/VP3	HPPPQILI[K_C13N15]
VP1	GLVLPGY[K_C13N15]
AAP	APTEWVIP[R_C13N15]
MAAP	LLGATSDEQSS[R_C13N15]

REFERENCE:

1. Lee, Z., et al., *Construction of an rAAV producer cell line through synthetic biology.* ACS Synth Biol, 2022. **11**(10): p. 3285-3295.
2. Lee, Z., et al., *Development of an inducible, replication-competent assay cell line for titration of infectious recombinant adeno-associated virus vectors.* Hum Gene Ther, 2023. **34**(3-4): p. 162-170.
3. Robinson, M.D., D.J. McCarthy, and G.K. Smyth, *edgeR: a Bioconductor package for differential expression analysis of digital gene expression data.* Bioinformatics, 2009. **26**(1): p. 139-140.
4. Subramanian, A., et al., *Gene set enrichment analysis: A knowledge-based approach for interpreting genome-wide expression profiles.* Proceedings of the National Academy of Sciences, 2005. **102**(43): p. 15545-15550.
5. Mootha, V.K., et al., *PGC-1alpha-responsive genes involved in oxidative phosphorylation are coordinately downregulated in human diabetes.* Nat Genet, 2003. **34**(3): p. 267-73.
6. Huang, D.W., B.T. Sherman, and R.A. Lempicki, *Systematic and integrative analysis of large gene lists using DAVID bioinformatics resources.* Nature Protocols, 2009. **4**(1): p. 44-57.
7. Sherman, B.T., et al., *DAVID: a web server for functional enrichment analysis and functional annotation of gene lists (2021 update).* Nucleic Acids Res, 2022. **50**(W1): p. W216-W221.
8. MacLean, B., et al., *Skyline: an open source document editor for creating and analyzing targeted proteomics experiments.* Bioinformatics, 2010. **26**(7): p. 966-968.
9. Lu, M., Z. Lee, and W.S. Hu, *Multi-omics kinetic analysis of recombinant adeno-associated virus production by plasmid transfection of HEK293 cells.* Biotechnol Prog, 2024. **40**(2): p. e3428.
